# Antitumor efficacy and potential mechanism of FAP-targeted radioligand therapy combined with immune checkpoint blockade

**DOI:** 10.1038/s41392-024-01853-w

**Published:** 2024-06-03

**Authors:** Liang Zhao, Yizhen Pang, Yangfan Zhou, Jianhao Chen, Hao Fu, Wei Guo, Weizhi Xu, Xin Xue, Guoqiang Su, Long Sun, Hua Wu, Jingjing Zhang, Zhanxiang Wang, Qin Lin, Xiaoyuan Chen, Haojun Chen

**Affiliations:** 1 Department of Nuclear Medicine and Minnan PET Center, Xiamen Key Laboratory of Radiopharmaceuticals, The First Affiliated Hospital of Xiamen University, School of Medicine, Xiamen University, Xiamen, China; 2 Departments of Diagnostic Radiology, Surgery, Chemical and Biomolecular Engineering, and Biomedical Engineering, Yong Loo Lin School of Medicine and College of Design and Engineering, National University of Singapore, Singapore, Singapore; 3 Clinical Imaging Research Centre, Centre for Translational Medicine, Yong Loo Lin School of Medicine, National University of Singapore, Singapore, Singapore; 4 Department of Radiation Oncology, Xiamen Cancer Center, Xiamen Key Laboratory of Radiation Oncology, The First Affiliated Hospital of Xiamen University, School of Medicine, Xiamen University, Xiamen, China; 5 Department of Colorectal Tumor Surgery, Xiamen Cancer Center, Xiamen Key Laboratory of Radiation Oncology, The First Affiliated Hospital of Xiamen University, School of Medicine, Xiamen University, Xiamen, China; 6 Department of Cardiothoracic Surgery, Zhongda Hospital, School of Medicine, Southeast University, Nanjing, China; 7 Nanomedicine Translational Research Program, NUS Center for Nanomedicine, Yong Loo Lin School of Medicine, National University of Singapore, Singapore, Singapore; 8 Department of Neurosurgery and Department of Neuroscience, Fujian Key Laboratory of Brain Tumors Diagnosis and Precision Treatment, Xiamen Key Laboratory of Brain Center, the First Affiliated Hospital of Xiamen University, School of Medicine, Xiamen University, Xiamen, China; 9 Institute of Molecular and Cell Biology, Agency for Science, Technology and Research (A*STAR), Singapore, Singapore; 10 Xiamen Key Laboratory of Rare Earth Photoelectric Functional Materials, Xiamen Institute of Rare Earth Materials, Haixi Institute, Chinese Academy of Sciences, Xiamen, China

**Keywords:** Drug development, Translational research

## Abstract

Radiotherapy combined with immune checkpoint blockade holds great promise for synergistic antitumor efficacy. Targeted radionuclide therapy delivers radiation directly to tumor sites. LNC1004 is a fibroblast activation protein (FAP)-targeting radiopharmaceutical, conjugated with the albumin binder Evans Blue, which has demonstrated enhanced tumor uptake and retention in previous preclinical and clinical studies. Herein, we demonstrate that ^68^Ga/^177^Lu-labeled LNC1004 exhibits increased uptake and prolonged retention in MC38/NIH3T3-FAP and CT26/NIH3T3-FAP tumor xenografts. Radionuclide therapy with ^177^Lu-LNC1004 induced a transient upregulation of PD-L1 expression in tumor cells. The combination of ^177^Lu-LNC1004 and anti-PD-L1 immunotherapy led to complete eradication of all tumors in MC38/NIH3T3-FAP tumor-bearing mice, with mice showing 100% tumor rejection upon rechallenge. Immunohistochemistry, single-cell RNA sequencing (scRNA-seq), and TCR sequencing revealed that combination therapy reprogrammed the tumor microenvironment in mice to foster antitumor immunity by suppressing malignant progression and increasing cell-to-cell communication, CD8^+^ T-cell activation and expansion, M1 macrophage counts, antitumor activity of neutrophils, and T-cell receptor diversity. A preliminary clinical study demonstrated that ^177^Lu-LNC1004 was well-tolerated and effective in patients with refractory cancers. Further, scRNA-seq of peripheral blood mononuclear cells underscored the importance of addressing immune evasion through immune checkpoint blockade treatment. This was emphasized by the observed increase in antigen processing and presentation juxtaposed with T cell inactivation. In conclusion, our data supported the efficacy of immunotherapy combined with ^177^Lu-LNC1004 for cancer patients with FAP-positive tumors.

## Introduction

The advent of immunotherapy has revolutionized modern oncology. Antibody-mediated blockade of programmed death 1 (PD-1) and its ligand (PD-L1) is increasingly considered as a cornerstone of cancer therapy. Antibodies that block the PD-1/PD-L1 interaction are used for treating various cancers, including melanoma, lung cancer, head and neck squamous cell carcinoma, nasopharyngeal cancer, colorectal cancer, liver cancer, gastric cancer, lymphoma, and urothelial cancer. If effective, PD-1/PD-L1 immune checkpoint blockade (ICB) can lead to a distinctive long-term response, whereby a small subset of patients experiences long-term remission. However, the observed response rates vary widely, ranging from 15 to 40% across different tumor types. Besides PD-1/PD-L1 checkpoint blockade, immunotherapies targeting other immune checkpoint factors, such as CTLA-4, LAG-3, and TIGIT, are currently being investigated in various clinical trials. However, like PD-1/PD-L1 antibodies, these treatments often exhibited limited effectiveness when used as standalone therapies. Given that PD-1/PD-L1 ICB is currently the most commonly used approach, researchers are exploring two primary strategies to boost its effectiveness. The first strategy involves identifying biomarkers to predict patient responses to ICB. However, the variability of these biomarkers across tumor types complicates their broader application. Another strategy is to combine immunotherapy with other traditional treatment methods, such as chemotherapy, radiotherapy, anti-angiogenic therapy, and targeted therapy. This combined approach has shown promise in enhancing the efficacy of PD-1/PD-L1 ICB. Consequently, the development of combination therapies aimed at enhancing the antitumor efficacy of ICB has become an area of intensive research.^1^

Radiotherapy, when combined with PD-1 or PD-L1 antibody treatment, has shown potential in boosting the infiltration of cytotoxic T-cells within the tumor microenvironment (TME) and attenuating immunosuppressive factors to improve antitumor efficacy.^2^ The advantages of this combination approach have been demonstrated in both preclinical and clinical studies.^2–4^ A pooled analysis of the PEMBRO-RT (Phase 2, NCT02492568) and MDACC (Phase 1/2, NCT02444741) trials, which included 148 patients, demonstrated a notable improvement in median overall survival.^3^ The combination of pembrolizumab, a PD-1 antibody, with radiotherapy resulted in a median overall survival of 19.2 months, compared to 8.7 months when pembrolizumab was used alone.^3^ However, treating widespread metastases or metastases near vital organs using external beam radiotherapy remains a challenge. Targeted radionuclide therapy offers a promising alternative to traditional radiotherapy as the tethering of radionuclides to molecules that specifically bind to diseased cells or tissues allows precise tumor irradiation.^5^ Moreover, targeted radionuclide therapy involves low-dose continuous radiotherapy, differing in its regulatory effect on the TME compared to high-dose external beam radiotherapy. Delivered intravenously, this treatment is particularly advantageous for patients with widespread metastases. To date, the main vectors used in radionuclide therapy target specific cancer types, such as ^177^Lu-PSMA-617 for metastatic prostate cancer and ^177^Lu-DOTATATE for neuroendocrine tumors.^5^ This tailored approach allows for the precise delivery of radiation to cancer cells, reducing damage to surrounding healthy tissues and enhancing outcomes for patients with metastatic tumors. However, both radiopharmaceuticals are suitable for specific cancer types, indicating a limited therapeutic range.

An emerging target of interest in cancer is fibroblast activation protein (FAP), which is widely expressed in cancer-associated fibroblasts (CAFs) across various tumor types, whereas it is expressed at low levels in normal tissues.^6^ This heightened expression is particularly evident in cancers that are characterized by strong desmoplastic responses, such as intrahepatic cholangiocarcinoma, colorectal cancer, pancreatic cancer, and breast cancer. FAP plays a notable role in promoting tumor growth, migration, and progression, with its overexpression being linked to poor prognosis in certain cancers. Thus, FAP-targeting radiopharmaceuticals (also known as fibroblast activation protein inhibitors [FAPIs]) are of particular research interest. As a pancancer-targeting molecule, FAPI-04/46 and OncoFAP have shown promise for various types of tumor PET imaging.^7–9^ However, the therapeutic applications of FAP-targeting molecules have been limited owing to their short retention times in tumor tissues.^10^ In our previous work, we demonstrated that modifying FAPI with the albumin binder Evans Blue (EB-FAPI, denoted as LNC1004) enhanced tumor uptake and retention, thus improving the antitumor efficacy of the therapeutic radionuclide ^177^Lu.^11,12^

Combining radionuclide-targeted therapy against pancancer targets with immunotherapy may be a promising treatment strategy for certain advanced tumors. Moreover, single-cell RNA-sequencing (scRNA-seq) technology offers a comprehensive view of cellular and molecular interactions at an unprecedented resolution.^13^ However, to date, no studies have reported the application of scRNA-seq in radionuclide-targeted or combined immunotherapies. Here, we investigated the therapeutic efficacy of ^177^Lu-LNC1004 in combination with anti-PD-L1 antibody in a preclinical setting. We pioneered the use of scRNA-seq for analyzing the changes within the TME and elucidated the underlying mechanisms of action of this combination treatment. Additionally, we assessed the safety and efficacy of ^177^Lu-LNC1004 in a small cohort of patients with various cancer types and analyzed the abundance of immune cell types among peripheral blood mononuclear cells (PBMCs) pre- and posttreatment.

## Results

### ^68^Ga/^177^Lu-LNC1004 exhibit pronounced uptake and prolonged retention in FAP-expressing tumor models

**Fig. 1:**
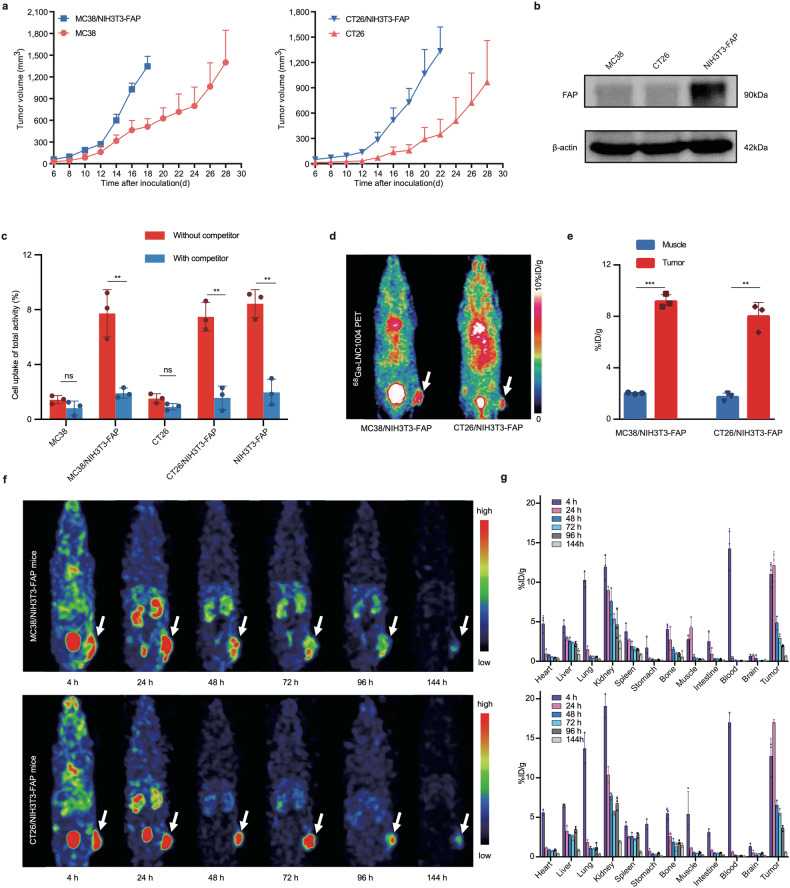
In vitro and in vivo evaluation of ^68^Ga/^177^Lu-labeled LNC1004**. a** Growth curves of tumors in mice following implantation of either MC38 or CT26 cells compared with those co-implanted with NIH3T3-FAP cells (*n* = 5/group). **b** FAP expression in MC38, CT26, and NIH3T3-FAP cells determined using western blotting. **c** Cell uptake assay of ^68^Ga-LNC1004 on MC38, CT26, NIH3T3-FAP, MC38/NIH3T3-FAP, and CT26/NIH3T3-FAP cells. This assay was complemented with a blocking experiment to validate specificity (*n* = 3/group). **d** Representative static PET images of ^68^Ga-LNC1004 in MC38/NIH3T3-FAP and CT26/NIH3T3-FAP tumor-bearing mice (*n* = 3/group). **e** PET quantification data for ^68^Ga-LNC1004 in MC38/NIH3T3-FAP and CT26/NIH3T3-FAP tumor-bearing mice (*n* = 3/group). **f**, **g** SPECT MIP images and biodistribution data of ^177^Lu-LNC1004 from 4 to 144 h after injection in mice with MC38/NIH3T3-FAP and CT26/NIH3T3-FAP tumor models (*n* = 3/group)

We observed that tumor volume was significantly larger in mice co-inoculated with NIH3T3-FAP fibroblasts and tumor cells than in controls inoculated with tumor cells alone (Fig. 1a). Furthermore, we did not observe any differences in body weight across the groups (Supplementary Fig. 1a). This observation was consistent with the results of prior studies involving B16, CT26, and 4T1 tumors mixed with NIH3T3 fibroblasts.^14–16^ Western blotting revealed that FAP was highly expressed in the NIH3T3-FAP cell line, whereas was not expressed in MC38 and CT26 cells (Fig. 1b). Using in vitro cell-based studies, we found that ^68^Ga-LNC1004 exhibited a strong binding affinity for FAP in NIH3T3-FAP cells, whether alone or cocultured with MC38 or CT26 cells (Fig. 1c). Moreover, we detected that unlabeled FAPI-46 effectively blocked the binding of ^68^Ga-LNC1004 to FAP in these cells. Of note, neither MC38 nor CT26 cells bound ^68^Ga-LNC1004. Immunohistochemistry (IHC) analysis of tumor tissues also indicated that FAP expression was predominantly observed on CAFs, rather than on the tumor cells (Supplementary Fig. 1b). In vivo bioluminescence imaging demonstrated that LUC-transfected NIH3T3-FAP cells coexisted with tumor cells within MC38 tumor tissue (Supplementary Fig. 2a). Furthermore, ^68^Ga-FAPI-46 PET imaging revealed no tracer uptake in MC38 tumors but positive uptake in MC38/NIH3T3-FAP tumor tissue (Supplementary Fig. 2b, c). We employed PET and SPECT imaging to assess the tumor uptake of ^68^Ga-LNC1004 and ^177^Lu-LNC1004 in vivo, respectively. We observed the intense and rapid uptake of ^68^Ga-LNC1004 in the two mixed tumor models, MC38/NIH3T3-FAP and CT26/NIH3T3-FAP, at 4 h post-injection (p.i.) (Fig. 1d). Quantification of data derived from small-animal PET showed that ^68^Ga-LNC1004 uptake in tumors was significantly higher than that in muscle tissues (Fig. 1e). Furthermore, SPECT imaging of both mixed tumor models demonstrated an intense tumor uptake of ^177^Lu-LNC1004 and high tumor-to-background contrast up to 96 h p.i. (Fig. 1f). We noticed that the biodistribution data corresponded well with the SPECT findings, demonstrating that the uptake of ^177^Lu-LNC1004 was the highest at 24 h p.i. Subsequently, we detected a gradual decrease in ^177^Lu-LNC1004 tumor uptake from 24 to 96 h p.i. (Fig. 1g), followed by a significant decrease at 144 h p.i.

### ^177^Lu-LNC1004 stimulation upregulates tumor PD-L1 expression both in vitro and in vivo

**Fig. 2:**
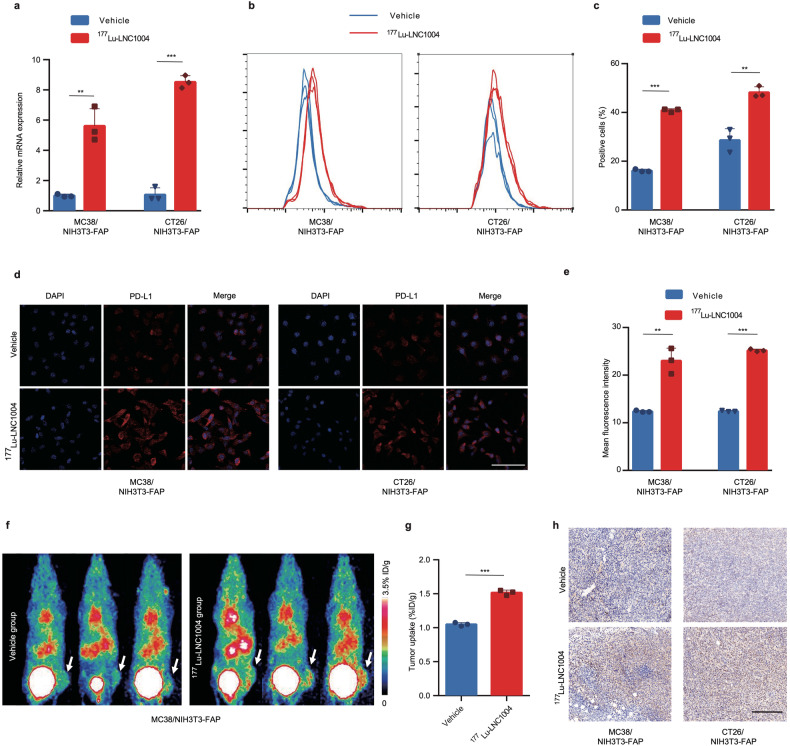
PD-L1 expression is significantly upregulated both in vitro and in vivo after treatment with ^177^Lu-LNC1004. **a** Bar plot derived from quantitative RT-PCR used to assess the mRNA levels of PD-L1 in MC38/NIH3T3-FAP and CT26/NIH3T3-FAP cells after 24 h stimulation with ^177^Lu-LNC1004 (*n* = 3/group). **b**, **c** Representative histograms and bar plot derived from flow cytometry showing the upregulation in PD-L1 expression after 24 h of stimulation with ^177^Lu-LNC1004 (*n* = 3/group). **d**, **e** Confocal images and bar plot derived from PD-L1 immunofluorescence staining indicate enhanced expression of PD-L1 after 24 h exposure to ^177^Lu-LNC1004 (n = 3/group). Scale bar: 100 μm. **f** Representative static PET maximum intensity projection (MIP) images of ^68^Ga-DOTA-SETSKSF in MC38/NIH3T3-FAP tumor-bearing mice (*n* = 3/group). **g** PET quantification data for ^68^Ga-DOTA-SETSKSF in MC38/NIH3T3-FAP tumor-bearing mice (*n* = 3/group). **h** Immunohistochemical staining of PD-L1 in tumor tissues. Scale bar: 200 μm

At the transcriptional level, we observed a greater than 5-fold upregulation of PD-L1 mRNA expression in MC38/NIH3T3-FAP and CT26/NIH3T3-FAP cells after 24 h of incubation with ^177^Lu-LNC1004 (Fig. 2a). Flow cytometry analysis revealed a notable increase in the proportion of PD-L1-positive cells after ^177^Lu-LNC1004 administration (Fig. 2b, c). In addition, to rule out the possibility of PD-L1 regulation by LNC1004 itself, the precursor of “cold” LNC1004, which was not radiolabeled with ^177^Lu, was co-incubated with tumor cells (Supplementary Fig. 3). We further confirmed this increase using immunofluorescence staining (Fig. 2d, e). In addition to the in vitro cell study, we evaluated alterations in PD-L1 expression in tumor models by employing a previously reported PD-L1-targeting radiotracer, ^68^Ga-DOTA-SETSKSF, for PET imaging of PD-L1 expression.^17^ We found that compared with the vehicle group, the ^177^Lu-LNC1004-treated group exhibited a significantly higher tumor uptake of ^68^Ga-DOTA-SETSKSF (Fig. 2f). Using PET quantitation analysis, we further validated that the tumor uptake of ^68^Ga-DOTA-SETSKSF in the ^177^Lu-LNC1004-treated group was significantly greater than that in the vehicle group (*P* < 0.001; Fig. 2g). Consistent with PET findings, IHC examination revealed enhanced PD-L1 expression in tumor tissues from the ^177^Lu-LNC1004 group (Fig. 2h).

### ^177^Lu-LNC1004, when combined with anti-PD-L1 immunotherapy, exhibits synergistic antitumor efficacy

**Fig. 3:**
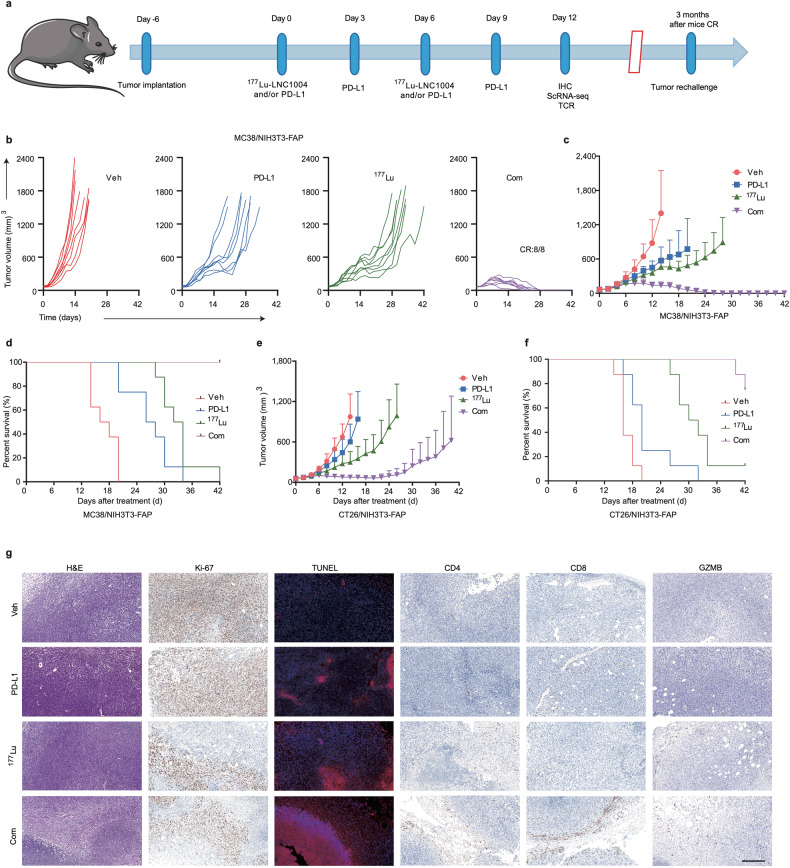
^177^Lu-LNC1004 radioligand therapy combined with anti-PD-L1 immunotherapy synergistically enhances antitumor efficacy**. a** Illustration of the therapeutic regimen and treatment timelines for mice bearing MC38/NIH3T3-FAP and CT26/NIH3T3-FAP tumor models (*n* = 8/group). **b** Individual tumor growth trajectories of MC38/NIH3T3-FAP tumor-bearing mice across diverse treatment groups. **c**, **d** Tumor growth and survival rate graphs for MC38/NIH3T3-FAP tumor-bearing mice in the four distinct treatment groups. **e**, **f** Tumor growth and survival rate graphs for CT26/NIH3T3-FAP tumor-bearing mice in the four different treatment groups. **g** Histological examination of resected tumor tissues from MC38/NIH3T3-FAP tumor-bearing mice, featuring hematoxylin & eosin (H&E) staining and immunohistochemical staining for Ki-67, TUNEL, CD4, CD8, and GZMB posttreatment. Scale bar: 200 μm

Given the upregulation of tumor PD-L1 expression by ^177^Lu-LNC1004 stimulation, we further explored the antitumor efficacy of a combination treatment including anti-PD-L1 antibody (αPD-L1) and ^177^Lu-LNC1004 radioligand therapy. The detailed therapeutic regimens for each group are shown in Fig. 3a. In the case of MC38/NIH3T3-FAP mixed tumor model, we found that tumor-bearing mice in the vehicle group showed rapid tumor growth, leading to total mortality by day 20 posttreatment (Fig. 3b–d). Although both the αPD-L1 and ^177^Lu-LNC1004-treated groups exhibited relatively slower tumor growth rates than those in the vehicle group, all mice died by days 34 and 42, respectively. We detected that the αPD-L1 + ^177^Lu-LNC1004 combination therapy group exhibited the most remarkable antitumor efficacy, with all mice (8/8) achieving a complete response (CR) by day 28 posttreatment. To further assess the persistence of immunological memory, we rechallenged these 8 CR mice with the same mixed cells (1 × 10^6^ MC38 and 2 × 10^6^ NIH3T3-FAP) in the left rear flank 3 months after achieving CR. Impressively, all rechallenged mice robustly rejected the reintroduced tumors. In the case of CT26/NIH3T3-FAP tumor-bearing mice, we determined that tumor growth among the different groups was similar to that observed for MC38/NIH3T3-FAP tumor models (Fig. 3e, f and Supplementary Fig. 4a). However, we found that only 3/8 mice in the αPD-L1 + ^177^Lu-LNC1004 combination group achieved CR. Moreover, we detected a slight decline in the body weight of mice in the ^177^Lu-LNC1004 and combination therapy groups, which was subsequently recovered (Supplementary Fig. 4b, c). H&E staining of the major organs on day 12 post-treatment in both tumor models revealed no discernible injury in any of the groups (Supplementary Fig. 4d, e). Furthermore, an assessment for potential long-term toxicity using H&E staining was performed 8 months after the treatment (Supplementary Fig. 5), which showed no significant signs of kidney toxicity in the group undergoing combination therapy. This finding provides a promising indication for the long-term safety profile of the combination treatment.

We further characterized the microenvironment of MC38/NIH3T3-FAP tumor models. In particular, using H&E and TUNEL staining, we detected pronounced cell necrosis and apoptosis, respectively, in the tumors of the combination therapy group (Fig. 3g). In addition, a lower percentage of Ki-67-positive cells in this group indicated reduced cellular proliferation. We also identified notable increases in the numbers of CD4- and CD8-positive T cells in the TME and the enhanced activity of granzyme B (GZMB) in this group, indicating a heightened antitumor immune response.

### scRNA-seq reveals cell types and intercellular communication within the TME

**Fig. 4:**
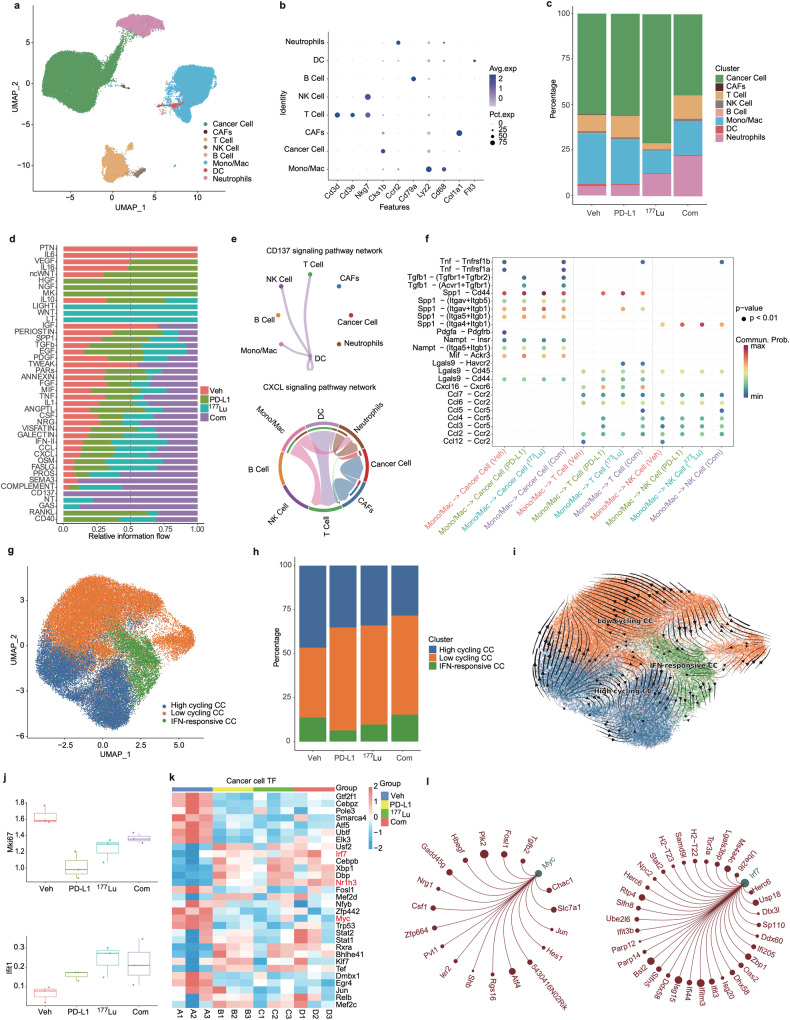
Cell type identification and cancer cell characterization in MC38/NIH3T3-FAP tumor models**. a** UMAP plots of all cells. **b** Dot plots reveal characteristic marker genes across different cellular fractions. **c** Bar charts comparing the major cellular lineages across various treatments. **d** Bar charts depict the varied contribution of pathways to cellular communication. **e** Interaction networks emphasize specific cell-to-cell interactions via the CD137 (top) and CXCL (bottom) pathways in the combination treatment group. **f** Highlighted ligand-receptor interactions from Mono/Mac with cancer cells, T-cells, and NK cells, as indicated by CellChat. **g** UMAP plots of cancer cells. **h** Bar charts show the distribution of tumor cell subpopulations. **i** RNA trajectory analysis reflects the evolutionary progression of tumor cells. **j** Box line plots representing mean Mki67 and Ifit1 expression in cancer cells. **k** SCENIC analysis delineating differences in AUC values of transcription factors (TFs). **l** Regulatory network diagram centered on TFs Myc and Irf7

We isolated single cells from MC38/NIH3T3-FAP mixed tumor tissues from various treatment groups. scRNA-seq analysis revealed eight predominant cell types in the tumor tissue: cancer cells, neutrophils, dendritic cells (DCs), monocytes and macrophages (Mono/Mac), B-cells, NK cells, T-cells, and CAFs (Fig. 4a, b and Supplementary Fig. 6a–f). Notably, we found that the proportions of T-cells, Mono/Mac, neutrophils, and cancer cells were substantially different between different treatment groups (Fig. 4c and Supplementary Fig. 6c). In addition, regarding the limited presence of CAFs in the scRNA-seq results, it is important to note that the CAF clusters identified are spontaneously generated by tumor tissues (which do not include NIH3T3-FAP cells), resulting in their limited proportion. Moreover, our scRNA-seq analysis did not encompass exogenously injected NIH3T3-FAP cells. Given that NIH3T3, mouse embryonic lung fibroblasts, are not normal diploid cells but rather aneuploid,^18,19^ they are likely to be filtered out as abnormal components during the single-cell sequencing process by the Cell Ranger program due to their excessive RNA content.

We identified distinct variations in cell signaling pathway activation across the four treatment groups, highlighting the potential core signaling pathways integral to the treatment response (Fig. 4d). Notably, we detected that the CD137 pathway was exclusively activated in the combination therapy group. This signaling was mainly implicated in the interplay network of DCs with T-cells, NK cells, and Mono/Mac, as well as between DCs (Fig. 4e). Such interactions are known to bolster antitumor immunity.^20^ In addition, we found that the CXCL signaling pathway was also highly active in the combination group (Fig. 4d). Both Mono/Mac and DCs demonstrated pronounced interactions with T-cells (Fig. 4e and Supplementary Fig. 7a–c) within the CXCL signaling pathway network, reinforcing their association with enhanced antitumor immunity.

We then assessed the alterations in ligand-receptor interactions to detect shifts in cell-to-cell communication across different treatment groups. In general, we noticed that the combination therapy group exhibited the most pronounced activation of signaling pathways, resulting in heightened inflammation and increased immune activity within tumors. Specific to CXCR signaling, using CellChat, we identified the ligand-receptor pairing of CXCL16-CXCR6 as a key signaling interaction in the combination group compared with the vehicle group (Supplementary Fig. 7d). This interaction plays a crucial role in facilitating the communication between DCs and T-cells. This observation was consistent with previous findings that CXCR6 expression is critical for the expansion of effector T-cells in the TME.^21,22^ In the combination group, the CCL5-CCR5 axis emerged as a notable signaling conduit from Mono/Mac cells to T-cells and NK cells (Fig. 4f). Interestingly, this axis has been implicated in the accumulation of cytotoxic T-cells and NK cells in tumors, thereby amplifying antitumor efficacy.^23^

### Combination treatment with ^177^Lu-LNC1004 and αPD-L1 antibody impedes malignant progression

We further explored the cancer cell populations in different groups. Upon reclustering of cancer cells, three distinct clusters emerged: high-cycling, low-cycling, and IFN-responsive cancer cells (Fig. 4g and Supplementary Fig. 8a). We further categorized these subclusters based on the expression levels of TPX2, CENPE, MKI67, and type I interferon-inducible gene (IFIT1).^14^ We observed that the combination therapy group exhibited the lowest proportion of high-cycling tumor cells, whereas the IFN-responsive cancer cell count was the highest (Fig. 4h). For a more comprehensive understanding, we employed the “RNA velocity” approach, which calculates trajectories on scRNA-seq UMAP plots.^24^ We predicted three distinct trajectories for all groups: high-cycling, low-cycling, or IFN-responsive cancer cell (Fig. 4i). Cell reprogramming analysis using RNA velocity revealed that high-cycling tumor cells have the potential to differentiate into low-cycling tumor cells. In addition, we found that IFN-responsive tumor cells predominantly originated from low-cycling tumor cells. Based on the reduction in the number of high-cycling tumor cells and increase in that of IFN-responsive tumor cells in the combination treatment group, we speculated that our combination therapy steered highly proliferative tumor cells towards a low-cycling status and further promoted the differentiation of low-cycling cells into IFN-responsive tumor cells, thus potentiating the immune response.

To elucidate the transcriptional programs activated in cancer cells in response to therapy, we performed differential expression analysis of the transcriptome and analyzed the AUC values of transcription factors (TFs) across the four distinct groups. Our primary focus was on the expression patterns that might influence cell proliferation and interactions with the immune system, as these are pivotal indicators of the therapeutic response. Notably, we detected that compared with the vehicle group, cancer cells from the other three groups (αPD-L1, ^177^Lu and combination treatment group) exhibited the downregulation of Mki-67 and upregulation of Ifit1 in response to therapy (Fig. 4j). Of note, the diminished expression of Mki-67 in cancer cells suggested decreased cell proliferation. Moreover, the expression of Ifit1 in tumors, which is induced by IFN, is associated with favorable outcomes in multiple cancer types.^25,26^ We further evaluated the SCENIC-identified tumor TFs based on their differences across the four treatment groups. The top 30 differentially expressed TFs are presented in a heatmap (Fig. 4k). In the combination treatment group, we detected a notable upregulation in Irf7 and Nr1h3 expression, whereas Myc expression was significantly downregulated. Myc regulates the Fosl1, Plk2, and Jun genes (Fig. 4l). Furthermore, Myc is essential for cancer cell proliferation and immune evasion.^27^ Irf7 regulates the expression of Ifit3, Ifitm3, Ifi44, and Ifit3b, all of which are members of the IFN-inducible gene family, implying their potential activation in response to interferon signaling (Fig. 4l). Nr1h3 regulates the levels of Mpeg1, Lst1, and Aif1 (Supplementary Fig. 8b), which are known to be expressed in macrophages. Nr1h3 has been reported to play a role in modulating macrophage activation and is associated with favorable prognosis in breast cancer.^28^ Taken together, these findings suggested that combination therapy with ^177^Lu-LNC1004 and αPD-L1 antibody may inhibit the malignant progression of cancer and prevent immune evasion.

### Combination therapy induces CD8^+^ T-cell expansion and Treg suppression

**Fig. 5:**
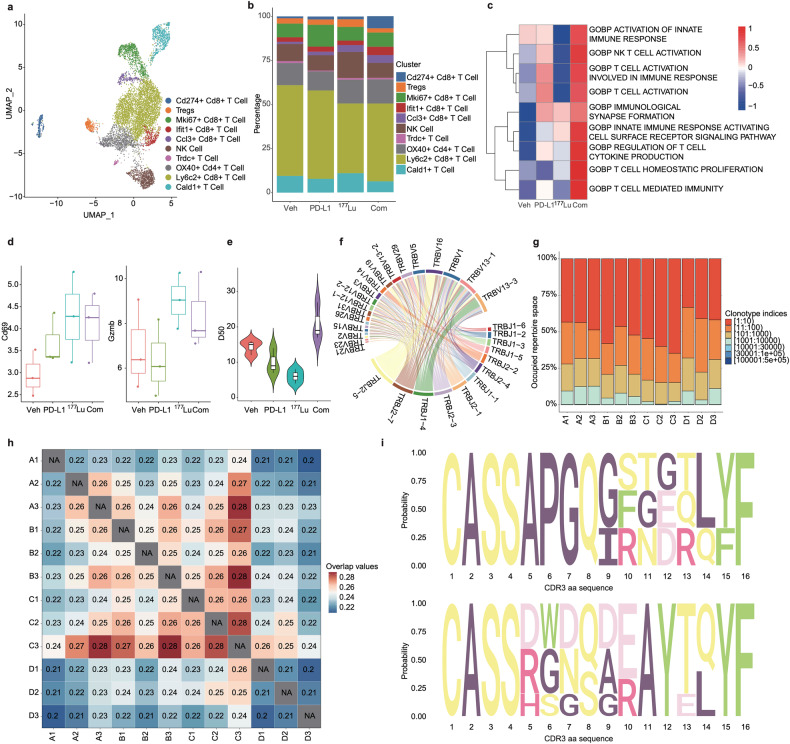
Differences in T-cell characteristics and TCRs across the four treatment groups**. a** UAMP plots of T-cell subclusters. **b** Bar charts showing the proportions of T-cell subclusters. **c** Heatmap illustrating the differentially active Gene Set Variation Analysis (GSVA)-enriched pathways associated with T-cells. **d** Box line plots of differential CD69 and GZMB expression in T-cells. **e** The D50 index, indicating TCR diversity, was analyzed and compared across the four treatment groups. **f** Chord plot illustrating the frequency of distinct V-J gene pairings in the combination treatment group. The V-J gene pairing frequencies in the other three treatment groups are shown in Supplementary Fig. 10c–e. **g** Graphical representation of clone frequency distribution based on the nucleotide CDR3 sequence rank (such as 1:10, 11:100, 101:1000, etc.). Each column represents the frequency distribution of T-cell clones within an individual tumor sample. The groups A-D correspond to the vehicle group, αPD-L1-treated group, ^177^Lu-LNC1004-treated group, and the combination therapy group with αPD-L1 and ^177^Lu-LNC1004, respectively. **h** Heatmap showing the overlap of immune repertoire clone types between the four treatment groups, as measured by the Jaccard index. The groups A–D correspond to the vehicle group, αPD-L1-treated group, ^177^Lu-LNC1004-treated group, and the combination therapy group with αPD-L1 and ^177^Lu-LNC1004, respectively. **i** Sequence logo diagrams present the CDR3 amino acid composition of the notably expanded TCR clonal family in the vehicle (upper) and combination treatment (lower) groups. These sequence logo diagrams for the αPD-L1-treated group and the ^177^Lu-LNC1004-treated group are shown in Supplementary Fig. 10f

We also explored the dynamics of tumor-infiltrating lymphocyte lineages in response to therapy. Upon reclustering the T/NK cells, we identified ten distinct clusters (Fig. 5a and Supplementary Fig. 9a). Consistent with IHC results (Fig. 3g), we noted a significant proliferation of total CD8^+^ T-cells in both the combination therapy and PD-L1-treated groups (Fig. 5b). In contrast, we detected a decrease in the total number of CD8^+^ T-cells in the ^177^Lu-LNC1004-treated group. We found that the number of Cd274^+^CD8^+^ T-cells (Cd274 is also known as PD-L1) was significantly increased in the combination group. Of note, Cd274^+^ T-cells have been reported to suppress the activity of effector T-cells in cancer, and have been associated with a worse prognosis in a cohort of patients with lung cancer not receiving immunotherapy.^29,30^ In contrast, patients with increased numbers of Cd274^+^CD8^+^ T-cells demonstrated a more favorable response to anti-PD-1 treatment.^30^ In addition, the interaction of Nampt with Itga5 and Itgb1 was uniquely observed in the combination therapy group (Supplementary Fig. 9b). This specific interaction occurred between Cd274^+^CD8^+^ T-cells and cancer cells, highlighting a distinct pathway in this therapeutic context. Notably, we found that the number of Ccl3^+^CD8^+^ T-cells was increased in both the combination and ^177^Lu-LNC1004 treatment groups. CD8^+^ T-cells can secrete C-C motif chemokine ligand 3 (Ccl3), also known as MIP-1α, subsequently amplifying T-cell recruitment and bolstering antitumor immunity.^31^ In addition, we observed a slight decrease in the proportion of Tregs in the combination treatment group, whereas an increase was observed in the ^177^Lu-LNC1004-treated group.

To further unravel the potential implications of these alterations in T-cell populations across the different treatment groups, we performed signaling pathway enrichment analysis. We accordingly identified different degrees of enrichment in each group, reflecting the varied therapeutic responses. Interestingly, most pathways observed in the combination treatment group, including “T-cell-mediated immunity” and “T-cell homeostatic proliferation”, were particularly enriched compared with those in other groups (Fig. 5c), suggesting enhanced antitumor efficacy. In contrast, we scarcely observed the enrichment of these pathways in the ^177^Lu-LNC1004-treated group. In addition, we detected augmented fractions of both CD69 and GZMB in the combination treatment group (Fig. 5d), both of which are markers of tissue-resident memory T-cells (Trm). Although CD69 is an indicator of early T-cell activation and Trm generation,^32^ it has also been associated with the exhaustion of tumor-infiltrating T-cells.^33^ Furthermore, increased GZMB expression has been implicated with bolstered T-cell-mediated killing of cancer cells.^34^

We next performed TCR-β sequencing of tumor tissues to further characterize the immune response. To avoid potential sample size biases, we employed the D50 metric to evaluate TCR-β diversity.^35^ Notably, we observed that the combination group exhibited increased TCR-β diversity compared with the other three groups (Fig. 5e). Such increased diversity has been reported to be associated with better clinical outcomes in patients undergoing ICB treatment.^35^ We then assessed the V-J gene utilization patterns of TRB among the four groups. We determined that all groups predominantly exhibited TRBV1 and TRBJ2-7 segments (Supplementary Fig. 10a, b). A similar finding was reported in a recent study in patients with esophageal squamous cell carcinoma who received combined radiotherapy and PD-1 blockade, in which TRBJ2-7 was reported to be the most prevalent V-J gene in intratumoral T-cells during treatment and in peripheral CD8^+^ T-cells both pre- and posttreatment.^36^ Furthermore, when examining the V-J pairing patterns across all samples (Supplementary Fig. 10c–e), we identified the TRBV16-TRBJ2-3 pairing as the most frequent in the vehicle group. However, this pairing was not as frequent during combination therapy (Fig. 5f), being replaced by the TRBV16-TRBJ2-5 pairing. Regarding clonal proportions, we found that the combination therapy group exhibited a decline in the frequencies of the top 10 clonal indices compared with the vehicle group (Fig. 5g). Conversely, we detected an increase in the 11-100 and 101-1000 clonal indices in the combination therapy group (Fig. 5g). We used the Jaccard index to measure the overlap of CDR3 sequences across samples from different groups.^37^ Notably, we observed a tendency towards reduced Jaccard similarity in the combination therapy group, both within the group and compared with the other three groups (Fig. 5h). This suggested that the TCRs in this group underwent more specific amplification, potentially enhancing their ability to suppress tumors. The sequence logo diagram offers visualization of the sequence site information, enabling a clear representation of sequence preferences. The results of the sequence logo diagram indicated significant variations in CDR3 sequence preference among the four groups (Fig. 5i and Supplementary Fig. 10f).

Taken together, our findings revealed the expansion and activation of CD8^+^ T-cells, reduction in the number of Tregs, and increased TCR diversity after treatment. These changes were associated with a positive response to the ^177^Lu-LNC1004 and anti-PD-L1 combination therapy.

### Combination therapy regulates macrophage polarization to enhance antitumor activity

**Fig. 6:**
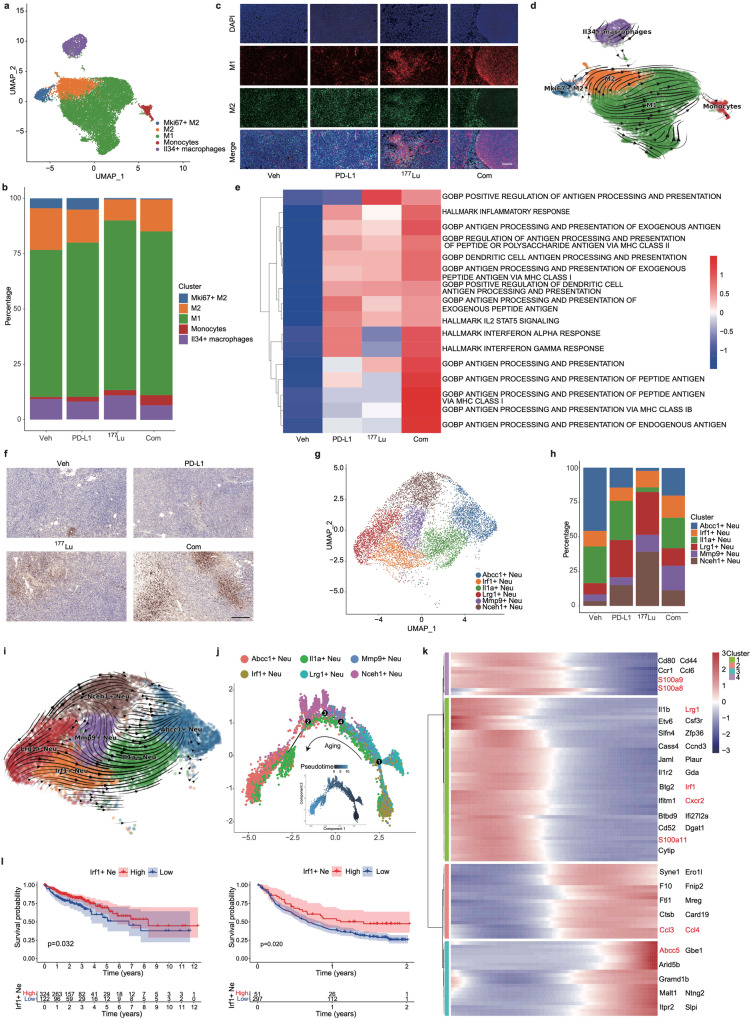
Comparative analysis of Mono/Mac and neutrophil characteristics across the four treatment groups. **a** UMAP plots of Mono/Mac subclusters. **b** Bar charts showing the distribution of Mono/Mac subpopulations. **c** Immunofluorescence staining of M1 and M2 macrophages in tumor tissues. Scale bar: 200 μm. **d** RNA trajectory analysis reflecting the evolutionary progression of Mono/Mac subclusters. **e** Heatmap illustrating the differential activity of GSVA-enriched pathways associated with Mono/Mac. **f** Immunohistochemical staining of Ly6G in tumor samples. Scale bar: 200 μm. **g** UMAP plots of neutrophil clusters. **h** Bar charts illustrating the distribution of neutrophil subpopulations. **i** RNA trajectory analysis reflects the evolutionary progression of neutrophil subtypes. **j** Monocle2 pseudo-temporal analysis reveals the evolutionary trends within neutrophil subclusters. **k** Heatmap of the top differentially expressed genes in neutrophils throughout pseudotime. **l** Kaplan–Meier survival plots for patients with colon (TCGA data, left) and bladder (IMgivo210 data, right) cancer. Comparative analysis of survival trajectories for IRF1^+^ neutrophil scores using the log-rank test

To gain further insight into the changes in immune cell subsets, we divided Mono/Mac cells into five distinct clusters: M1-like macrophages, M2-like macrophages, Mki67^+^ M2-like macrophages, monocytes, and Il34^+^ macrophages (Fig. 6a and Supplementary Fig. 11a). We observed a notable increase in the proportion of M1-like macrophages in the ^177^Lu-LNC1004 and combination therapy groups (Fig. 6b). In contrast, the numbers of M2-like and Mki67^+^ M2-like macrophages were decreased in both groups. We found that the changes in the expression of M1- and M2-like macrophage markers derived from the scRNA-seq data were consistent with the changes in their protein levels, as determined by immunofluorescence staining (Fig. 6c). Intriguingly, we detected that the proportion of Il34^+^ macrophages was reduced in the combination therapy group, whereas it was increased in the ^177^Lu-LNC1004 group. Il34^+^ macrophages are associated with immunosuppressive functions.^38^ Nevertheless, we noticed that the predominant trend within the combination treatment group was shifted towards bolstering antitumor immunity.

Utilizing RNA velocity, we elucidated the transcriptional trajectory of macrophages polarizing towards the two primary macrophage phenotypes: M1- and M2-like (Fig. 6d). Signaling pathway enrichment *via* Gene Set Variation Analysis (GSVA) revealed the notable upregulation of pathways associated with “antigen processing and presentation” in both the combination therapy and ^177^Lu-LNC1004-treated groups (Fig. 6e). However, Notable differences were detected regarding the “interferon alpha response” and “interferon gamma response” among groups. Both responses were upregulated in the combination treatment group, whereas suppressed in the ^177^Lu-LNC1004 group. These data suggested that combination treatment fosters a more immunologically active or “hot” TME. In contrast, both immune activation and suppression cooccurred in the ^177^Lu-LNC1004-treated group.

### Combination therapy promotes infiltration of Irf1^+^ neutrophils to suppress tumor growth

Given that neutrophils were the most abundant immune cells in the combination treatment group based on the scRNA-seq data (Fig. 4c), we performed IHC staining for the neutrophil marker Ly6G. Staining confirmed the notable increase in the number of neutrophils in the combination treatment group (Fig. 6f). A recent study has shown that elevated neutrophil counts following chemoradiotherapy are linked to improved progression-free survival after adjuvant immunotherapy in patients with lung cancer.^39^ Therefore, we further analyzed our neutrophil data and identified six distinct clusters: Abcc1^+^, Irf1^+^, Illa^+^, Lrg1^+^, Mmp9^+^, and Nceh1^+^ (Fig. 6g and Supplementary Fig. 11b). Notably, we found that the proportion of Irf1^+^ neutrophils in the combination treatment group exceeded those in the other three groups (Fig. 6h).

Using RNA velocity, we identified a dominant trajectory spanning all conditions. This trajectory extended from Lrg1^+^ and Irf1^+^ to Abcc1^+^ neutrophils (Fig. 6i). Pseudotime analysis using Monocle2 identified Lrg1^+^ and Irf1^+^ subclusters as the primary progenitors, evolving towards aging cell subclusters (Fig. 6j). In our analysis of gene expression dynamics, we identified four primary gene clusters that aligned with pseudotime progression (Fig. 6k). We further identified genes indicative of earlier cell states within these clusters, notably Irf1, Lrg1, and Cxcr2 (Fig. 6k). Aged neutrophils overexpress several chemokines including Ccl3, Ccl4, and Abcc5. A reduced proportion of aged neutrophils has been correlated with an enhanced immune response.^40^ In contrast, mature neutrophils predominantly express genes linked to granules, such as S100a8, S100a9, and S100a11, as well as Irf1, Lrg1, and Cxcr2. Notably, we observed that mature marker genes, particularly Irf1 and Lrg1, were distinctly positioned at the beginning of the developmental trajectory (Fig. 6j), showing enhanced expression in the combination treatment group. Granule release is crucial for the proinflammatory response, as recently reported.^41^ Another recent study asserted that Irf1 is pivotal in driving the activity of antitumor neutrophils, reporting that neutrophils lacking Irf1 expression undermine the efficacy of immunotherapy.^39^ Consequently, we hypothesized that an increase in the number of Irf1^+^ neutrophils could potentially hinder tumor progression.

To further ascertain the clinical relevance of Irf1^+^ neutrophils, we assessed the correlation between the numbers of Irf1^+^ neutrophils and clinical outcomes in patients with colon (TCGA data) and bladder (IMgivo210 data) cancer. Kaplan–Meier survival analysis indicated that a high proportion of Irf1^+^ neutrophils was associated with significantly better overall survival (*P* = 0.032) (Fig. 6l). The phase II IMgivo210 study revealed that patients with high counts of Irf1^+^ neutrophils experienced more favorable outcomes after immunotherapy than those with lower counts (Fig. 6l). In summary, our findings indicated that combination therapy enhanced the antitumor activity of neutrophils.

### Safety, tolerability, and preliminary efficacy of ^177^Lu-LNC1004 radioligand therapy in patients with refractory cancers

We conducted a preliminary clinical study (NCT05963386) to assess the safety, tolerability, and preliminary efficacy of ^177^Lu-LNC1004 in patients with advanced and refractory cancers. Between February and June 2023, five patients with different types of refractory cancer were enrolled in this study. The patient characteristics are summarized in Table 1. The median number of prior systemic treatments received by these patients was 4, ranging from 3 to 7. Two patients had a score of 3 according to the Eastern Cooperative Oncology Group (ECOG). All patients were administered a therapeutic dose of 3.3 ± 0.1 GBq/cycle. Because most patients were already in an advanced stage following the failure of multiple lines of treatment, four of these patients underwent only one cycle of treatment owing to their relatively poor health status. The remaining patient that was in a better condition underwent three cycles of treatment.

**Table 1:** Patient characteristics

Patient no.	Age (y)	Sex	Histology	Tumor sites (primary and metastatic)	Eastern Cooperative Oncology Group	No. of previous systemic therapies	^68^Ga-FAPI-46 (SUVmax baseline)	Status	Follow-up (d)
1	60	Woman	Breast Cancer	Lung, liver, bone, brain, soft tissue	3	3	12.5	Deceased	26
2	77	Man	Prostatic Cancer	Prostate, lymph nodes	1	3	19.7	Deceased	38
3	77	Man	Gastric Cancer	Gastric, lung, lymph nodes	0	4	16.2	Deceased	79
4	19	Woman	Breast Cancer	Lung, liver, lymph nodes	2	6	9.2	Deceased	60
5	60	Woman	Breast Cancer	Bone	3	7	24.7	Follow-up	115

**Fig. 7:**
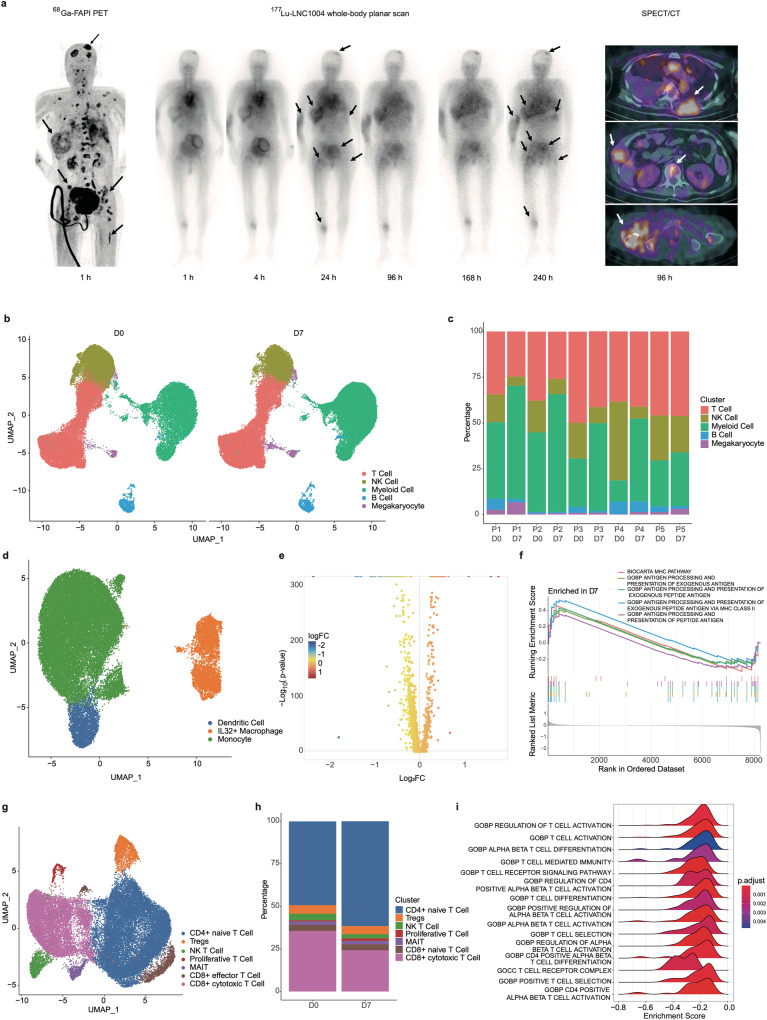
Single-cell RNA sequencing (scRNA-seq) of peripheral blood mononuclear cells (PBMCs) before and after treatment. **a** Representative images from PET, serial whole-body planar scan, and SPECT/CT scans of a patient (metastatic breast cancer) who received ^177^Lu-LNC1004 therapy. **b** UMAP plot of all cells color-coded according to their major cell types based on canonical markers. **c** Bar charts display the distribution of the principal cellular lineages in individual patients pre- and posttreatment. **d** UMAP plot showing the subpopulations of myeloid cells prior to and after treatment. **e** Volcano plots of differentially expressed genes within myeloid cells pre- and posttreatment. **f** Gene Set Enrichment Analysis (GSEA) of differentially expressed genes between pre- and posttreatment. **g** UMAP plot showing T-cell subpopulations pre- and posttreatment. **h** Bar charts showing variations in the proportion of T-cell subtypes across patients pre- and posttreatment. **i** GSEA of differentially expressed genes within T-cells pre- and posttreatment

There were no signs of any life-threatening adverse events (AE), clinically detectable pharmacological effects, or immediate AE-related significant changes in vital signs. In addition, none of the patients developed new hepatotoxicity or nephrotoxicity (Supplementary Table 1) following administration of ^177^Lu-LNC1004 radioligand therapy (RLT). However, one of the five (1/5) patients experienced grade 3 (G3) thrombocytopenia. Regarding the pharmacokinetic profile, we determined that ^177^Lu-LNC1004 exhibited a relatively high uptake in the blood pool at 1 h after administration, whereas a distinct uptake in tumor lesions was noted in a 24 h post-therapeutic, as revealed by a whole-body planner scan. Impressively, we could still observe an intense uptake of ^177^Lu-LNC1004 in most tumor lesions at 168 h after administration in all patients, indicating prolonged tumor retention. Representative images of the ^177^Lu-LNC1004 post-therapeutic whole-body scans at multiple time points are shown in Fig. 7a.

With a median follow-up period of 60 d (range: 26–115 d), one patient was still undergoing RLT, having completed three cycles of treatment. During follow-up, three patients died due to disease progression, whereas one patient died due to severe pneumonia caused by COVID-19. After treatment, four of the five patients reported relief of body pain symptoms and an improvement in quality of life. We observed a significant remission of widespread bone metastases in a patient with breast cancer (No. 5) after two cycles of ^177^Lu-LNC1004 treatment. Compared with the baseline scan, a follow-up ^68^Ga-FAPI-46 PET/CT demonstrated a considerable decrease in tumor size and reduced radiotracer uptake in most metastatic lesions (Supplementary Fig. 12).

To elucidate the transcriptional dynamics of PBMCs following ^177^Lu-LNC1004 treatment, we collected paired PBMC samples from five patients at baseline (D0) and 7 days after ^177^Lu-LNC1004 treatment (D7). After stringent quality control (Supplementary Fig. 13a), we classified cells into five principal cell types based on the expression of marker genes: T-cells, NK cells, monocytes, B-cells, and megakaryocytes (Fig. 7b and Supplementary Fig. 13b).

At 1 week after treatment, we observed a decline in the numbers of T-, NK, and B-cells in the majority of patients. Interestingly, opposite trend was detected in the myeloid cells, exhibiting an increase in their numbers (Fig. 7c). Delving deeper into the myeloid population, we identified three distinct subclusters: DCs, monocytes, and IL32^+^ macrophage (Fig. 7d). Despite the overall increase in the proportion of myeloid cells within the PBMC pool, the distribution among these three subclusters remained relatively consistent (Supplementary Fig. 14a, b). Using differential expression analysis, we identified 3041 upregulated and 5211 downregulated genes in myeloid cells after treatment (Fig. 7e, further details are shown in Supplementary Material 2). Gene Ontology (GO) enrichment analysis revealed that the genes upregulated in myeloid cells predominantly participated in several antigen processing and presentation pathways (Fig. 7f).

We then evaluated the proportions of various NK/T-cell subtypes (Fig. 7g and Supplementary Fig. 15). Notably, we observed an augmented representation of naive CD4^+^ T-cells, whereas a corresponding decrease in the percentage of CD8^+^ effector T-cells (Fig. 7h). These patterns observed after ^177^Lu-LNC1004 treatment in PBMCs mirrored the alterations observed in the proportion of CD8^+^ T-cells within the TME in our preclinical data, suggesting potential immunosuppressive shifts. Furthermore, GO analysis suggested the negative regulation of multiple T-cell activation pathways in PBMCs (Fig. 7i), in consistency with the results from tumor tissues in the preclinical study. Together with the previously described enhancement in antigen processing and presentation pathways, this finding suggested the presence of inhibitory mechanisms possibly involving immune checkpoints that hinder the proliferation and effector functions of T-cells.

## Discussion

Given the promising outcomes of combining radiotherapy and ICB, there is a growing interest in exploring targeted radionuclide therapy as a potential enhancer of the ICB efficacy.^42–44^ In this study, CR was achieved in the tumor-bearing mice treated with a combination of ^177^Lu-LNC1004 and anti-PD-L1 antibody. IHC staining and scRNA-seq revealed the reprogramming of the TME toward antitumor immunity in the combination treatment group, in contrast to TMEs in the vehicle and ^177^Lu-LNC1004/αPD-L1 monotherapy groups.

Of note, FAP is widely expressed in CAFs in various epithelial cancers, making it an attractive target for cancer theragnostics.^45^ To further enhance antitumor efficacy, chemically modified FAPIs were conjugated with the albumin binder Evans blue (EB) for improved tumor uptake and retention.^11,12^ However, a significant hurdle in advancing FAP-targeted theragnostics is the absence of suitable preclinical models that accurately mimic the TME, which is characterized by elevated FAP expression in CAFs. For example, the level of FAP expression in established tumor models, including MC38, CT26, LLC, and 4T1 tumor-bearing mice, is relatively low.^46^ However, combining NIH3T3-FAP cells with tumor cells has been demonstrated as a feasible method for establishing FAP-positive tumors.^15,47^ Compared with natural MC38 and CT26 tumors, tumor models exhibited a faster growth rate when mixed with NIH3T3-FAP cells. MC38/NIH3T3-FAP and CT26/NIH3T3-FAP tumors exhibited a high uptake of ^68^Ga-LNC1004 both in vitro and in vivo. Furthermore, in vivo SPECT imaging and biodistribution studies revealed the high retention of ^177^Lu-LNC1004 in both MC38/NIH3T3-FAP and CT26/NIH3T3-FAP tumors over extended periods. In a comprehensive study involving 359 tumor samples in 16 types, it was found that FAP expression was predominantly confined to CAFs in the stroma.^48^ Notably, tumor cell staining for FAP was more prevalent in sarcomas and mesotheliomas, and occasionally observed in various epithelial tumors.^48^ This evidence suggests that our mouse models, where fibroblasts are co-implanted with FAP-negative tumor cells, are able to partially mimic the TME observed in human solid tumors. The primary objective of our preclinical data is to propose an alternative and novel therapeutic strategy, rather than to precisely predict treatment efficacy with the same therapy in human malignancies.

Preclinical studies have demonstrated that radiation upregulates the expression of PD-L1 via the JAK/STAT pathway.^49,50^ Herein, we found that ^177^Lu-LNC1004 effectively upregulated PD-L1 expression, both in vitro and in vivo. Given that the interaction between PD-1 and PD-L1 is a primary mechanism for cancer immune evasion, combining ^177^Lu-LNC1004 with anti-PD-L1 immunotherapy could potentially amplify antitumor immunity. We observed that ^177^Lu-LNC1004 significantly inhibited tumor growth in both MC38/NIH3T3-FAP and CT26/NIH3T3-FAP tumor-bearing mice. Notably, combination therapy with ^177^Lu-LNC1004 and PD-L1 blockade synergistically enhanced therapeutic efficacy. Interestingly, all MC38/NIH3T3-FAP tumors were completely eradicated, with mice subsequently demonstrating a 100% tumor rejection rate upon rechallenge. Body weight measurements and histological examinations further supported the safety profile of this combination regimen. H&E and TUNEL staining of tumor tissues revealed pronounced necrosis and apoptosis in the combination therapy group, with IHC further confirming superior antitumor responses, including decreased tumor cell proliferation and enhanced T-cell infiltration.

To obtain a more comprehensive view, we employed scRNA-seq to analyze the changes in cancer and immune cell transcriptomes in response to therapy. Consistent with IHC findings, scRNA-seq revealed an increase in the number of T-cells and a decrease in that of tumor cells in the combination therapy group. The CD137 and CXCL signaling pathways were particularly upregulated, representing the interaction of Mono/Mac and DCs with T-cells. Analysis of TFs revealed the upregulation of Irf7 and Nr1h3, and downregulation of Myc, suggesting enhanced immune activation and hindered oncogenesis. In addition, we observed a reduction in the number of high-cycling tumor cells, whereas an increase in that of IFN-responsive tumor cells was detected. An increase was also noted in the number of M1 macrophages, whereas that of M2 macrophages was decreased, creating a favorable environment for T-cell activation. Signaling pathway enrichment analysis highlighted the enhanced immunological activity, particularly regarding antigen processing and presentation, in the combination treatment group. Furthermore, enhanced T-cell activation and an increased proportion of total and antitumor T-cells was also observed in the combination group. TCR-β sequencing indicated a higher diversity with more specific amplification in the combination group, which contributed to antitumor immunity.

In addition to T-cell, DC, and macrophage subclusters, neutrophils have recently garnered increasing attention in cancer research.^39,51,52^ Hirschhorn et al. reported that neutrophils were recruited to target and eliminate antigen loss variants, thus complementing T-cell-mediated tumor destruction.^52^ Gungabeesoon et al. highlighted that a therapy-induced IRF1 neutrophil response promoted tumor inhibition.^39^ Consistent with these studies, our data showed a significant increase in the numbers of total and IRF1 neutrophils in the combination therapy group. Furthermore, we found that higher levels of IRF1 in neutrophils correlated with better prognosis in patients with colon and bladder cancer. However, given the heterogeneous and multifaceted nature of neutrophils in cancer, with some studies indicating their association with immunosuppression,^53,54^ our results suggest that particular subgroups of neutrophils might enhance immunotherapy responses. This aspect should be interpreted with caution.

These encouraging preclinical results motivated us to perform a small-scale clinical trial of the ^177^Lu-LNC1004 radiopharmaceutical to translate our preclinical data. None of the enrolled patients experienced severe adverse events after ^177^Lu-LNC1004 administration, despite their deteriorating condition after multiple treatment lines. Posttherapeutic whole-body planner scan showed considerable uptake of ^177^Lu-LNC1004 in most tumor lesions, even at 240 h (10 d) after administration. After ^177^Lu-LNC1004 treatment, four patients reported an improvement in their symptoms (e.g., relieved bone and abdominal pain), with one patient noting a significant reduction in pelvic bone lesions. To investigate the posttreatment immune response, we collected PBMCs from each patient for scRNA-seq. Consistent with preclinical data from tumor tissues, we noted an activation of antigen processing and presentation pathways. However, we also observed a reduction in the number and activity of T-cells. Through the killing of malignant cells, radiation converts the TME into a highly immunogenic vaccine-like entity by prompting antigen release following cancer cell death. This allows antigen-presenting cells to activate NK and T-cells that target the remaining tumor cells.^55^ However, radiation may also boost PD-L1 expression in tumor cells, promoting immune evasion.^49,50,56^ Furthermore, an increase in the numbers of immunosuppressive populations may also hinder antitumor immunogenicity. This juxtaposition of augmented antigen processing and presentation with inactivated T-cells underscores the significance of overcoming immune evasion. Our findings supported the development of a combination therapy using anti-PD-L1 and ^177^Lu-LNC1004 for refractory cancers with positive FAP expression.

In most studies evaluating combination regimens of radiotherapy and immunotherapy, only a single or few specific sites are subjected to irradiation. This undermines the notion of heterogeneity among distinct lesions even in the same patient. Whereas, irradiating multiple or all lesions can amplify the synergistic effects of combined therapy with ICB.^57^ However, delivering external radiation to all metastatic lesions in patients with widespread metastases may not be feasible. Given the intense uptake of ^177^Lu-LNC1004 in various types of cancers, its combination with an anti-PD-L1 antibody may offer a suitable treatment alternative for patients with advanced and refractory disease.

The current study had several major limitations. First, FAP-positive CAFs constitute only a subset of the total CAFs in cancers. Preclinical tumor models derived from the co-injection of NIH-3T3-FAP cells with tumor cells only mimic parts of FAP high-expression clinical tumors. However, high levels of FAP expression have been documented across multiple human tumor types, as evidenced by IHC and FAPI PET/CT studies.^7,8,48^ This approach, while not representing the complete TME, provides valuable insights into FAP-overexpressing conditions. Moreover, the TME of subcutaneous tumors differs from that of spontaneous colon cancers to some degree. Therefore, exploring a spontaneous colon cancer model or an orthotopic colon cancer model would offer a more clinically relevant setting, which warrants further investigation. Second, obtaining tumor tissues from patients with advanced disease before and after ^177^Lu-LNC1004 therapy was difficult owing to their poor health status. Therefore, PBMCs were used as alternative samples for scRNA-seq. Moreover, we also collected tissues from tumor models as part of our preclinical study and subject them to scRNA-seq. Third, the number of patients who underwent ^177^Lu-LNC1004 radioligand therapy was small; the recruitment of more patients is still underway. Because of this, the results of therapeutic efficacy, including objective response and disease control rates, were not reported in this study. Fourth, combination therapy with ^177^Lu-LNC1004 and ICB was not recommended for these patients due to their poor overall condition. However, a similar therapeutic approach, combining other therapeutic radiopharmaceuticals (e.g., ^177^Lu-PSMA-617 for prostate cancer and ^177^Lu-DOTATATE for neuroendocrine tumor) and ICB, has demonstrated a safe profile and encouraging antitumor activity in recent studies.^44,58^ Further, one must note that the interpretation of IHC staining is often subjective and primarily serves to corroborate general trends or other observational findings.

In conclusion, our preclinical data suggested that ^177^Lu-LNC1004 can amplify the antitumor efficacy of ICB. Furthermore, preliminary clinical data indicated that ^177^Lu-LNC1004 is a safe and well-tolerated therapeutic regimen with encouraging antitumor activity. Our data foster further exploration of the synergy between ^177^Lu-LNC1004 and immunotherapy in patients with advanced and refractory disease, particularly in those with FAP-positive tumors.

## Materials and methods

### General

All chemicals were obtained from Nanchang Tanzhen Biological Technology Co. Ltd. (Jiangxi, China). The S-Cyclo(ETSK)-SF-NH2 (SETSKSF) PD-L1 probe was synthesized following previously described methods.^17^ High-purity radionuclide ^177^LuCl_3_ in 0.04 M HCl (3.9 GBq in 97 μL) was purchased from ITG (Munich, Germany). The radiolabeling process for FAPI-46, LNC1004, and SETSKSF variants with ^68^Ga or ^177^Lu closely followed previously reported protocols.^11,12,17^ The labeling efficiency and radiochemical purity were tested using a radio-TLC scanner (MSFC1-00220, Eckert & Ziegler, Germany), Dionex Ulti-Mate 3000 high-performance liquid chromatography (HPLC; Thermo Scientific, Waltham, MA, USA), an SPD-20A UV detector (*λ* = 254 nm), and an Elysia Raytest Gabi Star γ-radiation detector. Radioactivity was measured using a γ-counter (WIZARD 2480; PerkinElmer, Waltham, MA, USA) and CRC-25R dose calibrators (CAPIN-TEC Inc., USA). InVivoMab anti-mouse PD-L1 mAb was purchased from BioXCell (Cat. No. BE0101).

### Cell culture and establishment of tumor models

The murine colon adenocarcinoma cell lines MC38 and CT26 were procured from the Chinese National Infrastructure of Cell Line Resource. Stably transfected NIH3T3-FAP cells were kindly provided by Dr. Kai Cheng.^59^ C57BL/6 and BALB/c mice, both 6-weeks-old, were originally sourced from Beijing Vital River Laboratory Animal Technology Co., Beijing, China. All animal care and experimental protocols were reviewed and sanctioned by the Animal Care and Use Committee of the Xiamen University Laboratory Animal Center under approval number XMULAC20200140. For tumor implantation, the right rear flanks of either C57BL/6 or BALB/c mice were injected with a 100-μL suspension of tumor cells (MC38 or CT26, 1 × 10^6^ cells) with/without fibroblasts (NIH3T3-FAP, 2 × 10^6^ cells). This procedure was conducted in accordance with previous reports.^14,15,47^ All protocols involving animal care and experiments were thoroughly reviewed and approved by the Animal Care and Use Committee of the Xiamen University Laboratory Animal Center (approval number XMULAC20200140).

### In vitro studies

The cells were plated in 24-well plates with a medium containing 10% FBS and were allowed to grow until they reached an approximate confluence of 80%. Prior to experiments, the medium was replaced with fresh serum-free medium. Either ^68^Ga-LNC1004 alone or ^68^Ga-LNC1004 combined with 11.3 nmol of non-radioactive FAPI-46 (for the blocking experiment) was added to the wells and incubated for 1 h. Following incubation, the cells were lysed using 0.5 mL of 1 M NaOH, and the radioactivity was measured using a WIZARD 2480 γ-counter from PerkinElmer, Waltham, MA. Each experiment was performed in triplicate. The detailed WB, qPCR, flow cytometry, IHC, and immunofluorescence protocols are provided in Supplementary Material 1.

### Small-animal PET, SPECT imaging, biodistribution studies, and in vivo bioluminescence imaging

For the PET imaging studies, mice (three per group) were each administered roughly 7.4 MBq of ^68^Ga-FAPI-46, ^68^Ga-LNC1004, or ^68^Ga-DOTA-SETSKSF via intravenous injection. Imaging was conducted 1 or 4 h post-injection, with a 5-min static PET scan. The data were reconstructed using a 3D OPMAP 256.pPetRcn protocol from Siemens (Siemens Preclinical Solution, Germany), and the results were then transformed into %ID/g images. SPECT imaging was performed on mice bearing ^177^Lu-LNC1004 (three per group). Imaging sessions spanned from 4 to 144 h after injection with 18.5 MBq of the radiotracer. The imaging was conducted using a four-head multiplexing multi-pinhole camera (Mediso, Budapest, Hungary; window width: 20%, matrix: 256 × 256, medium zoom). SPECT data were reconstructed iteratively with software (Tera-Tomo; Mediso) using ^177^Lu γ-energies of 112.9 and 208.4 keV.

For studying biodistribution, mice were injected with 0.74 MBq of ^177^Lu-LNC1004. The mice were euthanized at predetermined time intervals ranging from 4 to 144 h post-injection. The tumors, as well as major organs and tissues, were then excised, weighed, and their radioactivity levels were measured. The data obtained were standardized and presented as a percentage of the injected dose per gram (%ID/g), taking into account 1% of the total counts. The details of the in vivo bioluminescence imaging method is shown in Supplementary Material 1.

### In vivo therapy regimen

Once the tumor volume reached approximately 50 mm³, mice bearing either MC38/NIH3T3-FAP or CT26/NIH3T3-FAP tumors were randomly assigned to various treatment groups (n = 8/group) (Fig. 3a): group A was treated with a vehicle; group B was treated with 18.5 MBq of ^177^Lu-LNC1004 intravenously (i.v.) on days 0 and 6; group C received anti-PD-L1 mAb intraperitoneally (i.p.) at a dose of 10 mg/kg on days 0, 3, 6, and 9; group D received combined therapy, consisting of 18.5 MBq of ^177^Lu-LNC1004 via i.v. and 10 mg/kg of anti-PD-L1 mAb via i.p. This combination regimen was based on our previous experience with the combination of ^177^Lu-EB-RGD (the EB conjugated integrin α_v_β_3_-targeted radiopharmaceutical) and anti-PD-L1 antibody.^42^ The tumor volumes and body weights of the mice were closely monitored every 2 days. If the tumor volume surpassed 1500 mm³, weight loss exceeded 15%, or abnormal behavior indicating pain or unease was observed, the respective mice were euthanized. Tumor tissues were analyzed using IHC for common markers and H&E staining on day 12 after treatment. Subsequently, tumor tissues from MC38/NIH3T3-FAP tumor models were subjected to scRNA-seq and TCR-β sequencing (details are provided in Supplementary Material 1).

### Analysis of publicly available data

We obtained colon cancer data from TCGA (https://xena.ucsc.edu/) and bladder cancer immunotherapy data from IMvigor210. We performed Kaplan–Meier (K-M) analysis to obtain survival curves for the IRF1+ neutrophil score, which was based on the IRF1 neutrophil hub gene set (which includes SLFN5, RNASEL, IRF1, S100A8, and IFI47) obtained via ssGSEA enrichment analysis.

### Clinical trial overview

This single-center, open-label, non-randomized investigator-initiated trial (IIT) was conducted at The First Affiliated Hospital of Xiamen University, China. The clinical study was registered at ClinicalTrials.gov under the identifier NCT05963386. The Clinical Research Ethics Committee of the First Affiliated Hospital of the Xiamen University approved this study, and all participants provided written informed consent.

### Patient selection

The inclusion criteria were adult patients (>18 years) with (1) histologically confirmed metastasized solid cancer; (2) unresectable tumors; (3) disease progression despite multiple therapies; and (4) tumor lesions showing significant radiotracer uptake on ^68^Ga-FAPI-46 PET/CT (defined as a maximum standardized uptake value ≥ 10 in more than 50% of tumor lesions). The exclusion criteria were as follows: (1) serum creatinine level >150 μmol/L; (2) hemoglobin level <8.0 g/dL; (3) white cell count <2.0 × 10^9^/L; (4) platelet count <50 × 10^9^/L; (5) total bilirubin level >3 times the upper limit of the normal range and serum albumin level <2.0 g/dL; (6) cardiac insufficiency, including carcinoid heart valve disease, severe allergy, or hypersensitivity to radiographic contrast material; (7) claustrophobia; and (8) pregnancy or breastfeeding.

### ^177^Lu-LNC1004 administration, scintigraphy imaging, SPECT/CT imaging, and clinical follow-up

^177^Lu labeling of LNC1004 was performed using above mentioned methods (see “General”). Furthermore, the samples were tested for quality, endotoxins, and sterility. Radiochemical purity, determined via thin-layer and high-performance liquid chromatography, was consistently above 98%. Prophylactic ondansetron and intravenous hydration were administered before the ^177^Lu-LNC1004 infusion. Patients underwent up to three treatment cycles, with monitoring of symptoms and vital parameters throughout. The details of scintigraphy imaging, SPECT/CT imaging, and clinical follow-up are shown in Supplementary Material 1. The PBMCs collected from patients were subjected to scRNA-seq pre- and post-treatment to observe alterations in the immune response.

### Statistical analysis

All statistical analyses were conducted using SPSS version 22.0 (IBM, Armonk, NY). One-way analysis of variance and the Student’s *t* test were used to compare means. Results with *P* value of <0.05 were considered statistically significant.

### Supplementary information

SUPPLEMENTARY MATERIAL 1

SUPPLEMENTARY MATERIAL 2

## Data Availability

All data associated with this study are present in the manuscript or Supplementary Materials. The original transcriptome data are available on the GSA Web site (https://ngdc.cncb.ac.cn/gsa/), under the project number CRA013101 (mouse tumor tissue) and HRA005996 (patients’ PBMC).
