# Supplementary material for: Antitumor efficacy and potential mechanism of FAP-targeted radioligand therapy combined with immune checkpoint blockade
Source: Signal Transduct Target Ther. 2024 Jun 3;9:142. doi: 10.1038/s41392-024-01853-w (PMC11144707; doi:10.1038/s41392-024-01853-w)
Supplement: Supplementary file 1 — SUPPLEMENTARY MATERIAL 1 [file 41392_2024_1853_MOESM1_ESM.docx]

**Supplementary Materials for**

**Antitumor efficacy and potential mechanism of FAP-targeted radioligand therapy combined with immune checkpoint blockade**

**Running Title:** FAP-targeted radioligand therapy with ICB

**Authors:** Liang Zhao^1^^,2,3†^, Yizhen Pang^1,2,3†^, Yangfan Zhou^1,4†^, Jianhao Chen^1,5†^, Hao Fu^1^, Wei Guo^1^, Weizhi Xu^1^, Xin Xue^6^, Guoqiang Su^5^, Long Sun^1^, Hua Wu^1^, Jingjing Zhang^2,3,7^, Zhanxiang Wang^8^, Qin Lin^4^*,Xiaoyuan Chen^2,3,7,9*^, Haojun Chen^1,10^*

^†^These authors contributed equally to this work.

*Corresponding author(s):

Email: [leochen0821@foxmail.com](mailto:leochen0821@foxmail.com) (H.C.); [chen.shawn@nus.edu.sg](mailto:chen.shawn@nus.edu.sg) (X.C.); linqin05@163.com (Q.L.).

**This PDF file includes:**

Materials and Methods

Supplementary Figures S1 to S15

Supplementary Table S1

**Supplemental methods**

*WB,* *qPCR, flow cytometry, IHC, and immunofluorescence*

Proteins were extracted with lysis buffer (150 mM NaCl, 50 mM Tris-HCl [pH 8.0], 1mM EDTA and 1% of the protease inhibitor and the phosphatase inhibitor). Approximately 20 μg of total protein per sample was separated by SDS-PAGE and transferred to a PVDF membrane (Millipore). The membranes were pre-incubated with 5% skimmed milk in TBST for 1 h, followed by incubation with mouse FAP antibody (ABclonal, Cat. No. A23789). Membranes were washed with TBST three times and incubated with horseradish peroxidase-labelled secondary antibody, which was detected using an enhanced chemiluminescence detection system (CLINX, ChemiScope 6200).

The cells were incubated with/without ^177^Lu-LNC1004 (3.7 MBq/mL) or LNC1004 (0.1 μg/mL) for 24 hours in serum-free medium and then were performed qPCR and flow cytometry. For qPCR, total RNA was isolated from tumor cells using the SteadyPure Quick RNA Extraction Kit (Accurate Biology, AG21023), following the manufacturer’s instructions. Subsequently, cDNA synthesis was performed through reverse transcription with the FastKing gDNA Dispelling RT SuperMix for RT-PCR from TIANGEN. For RT-PCR gene expression analysis, iScript and SsoAdvanced SYBR Green SuperMix by TIANGEN were employed, utilizing the ABI StepOne Plus Real-Time PCR system. The amplification of specific transcripts was verified by analyzing the melting curve profiles produced at the conclusion of the PCR program. The expression levels of the target genes were normalized to the housekeeping gene, b-actin, and quantified using the comparative cycle threshold method (2–ΔΔCt). Results were visualized through a bar plot generated with GraphPad Prism 8.0 software. All measurements were conducted in triplicate.

Regarding the flow cytometry, viable cells were counted and resuspended in cell staining buffer at a concentration of 5–10 × 10^6^ cells/mL. Next, 100 µL/tube of cell suspension (5–10 x 10^5^ cells/tube) was distributed into 2-mL plastic tubes. Fc receptors were then blocked by pre-incubation in 1 µg of purified anti-mouse CD16/CD32 mAb (BD PharmingenTM, Cat. No. 553141) per 10^6^ cells in 100 µL at 4 ºC for 5 min. Then, 100 μL of cells per tube were incubated with fluorescent mAbs specific to mouse PD-L1 (BD PharmingenTM, Cat. No. 564716) for 30 min at 2 –8 °C. The tubes were then washed once in 2 mL of stain buffer and centrifuged at 350 × g for 5 min. The cells were analyzed and data acquired using the BD FACSCanto II Flow Cytometer.

For immunofluorescence staining, tumor cells were seeded in confocal dishes and incubated for 24 hours with or without ^177^Lu-LNC1004 at a concentration of 3.7 MBq/mL. The samples were then fixed with 4% paraformaldehyde for 10 minutes. Subsequently, the cells were washed thrice with PBS and blocked with 10% goat serum for 30 minutes to minimize nonspecific binding. The cells were then incubated overnight with the primary antibody, anti–PD-L1 (Abcam, ab213480), at a dilution of 1/200, followed by three washes with PBS. Afterward, the cells were stained with the secondary antibody, DyLight 550–conjugated IgG, for one hour and washed three times with PBS. Nuclei were stained blue using DAPI (Biosharp, Cat. No. BL105A). The stained images were analyzed using ImageJ 7.0 software, with relative quantitation of immunofluorescent images performed according to our previously study.

For histological analysis, tissue specimens underwent a series of preparations. They were fixed in 10% buffered formalin, dehydrated through a series of ethanol washes, embedded in paraffin, and subsequently stained using Haematoxylin and Eosin (H&E) or Immunohistochemistry (IHC). The paraffin-embedded samples were dewaxed in ethanol and subjected to antigen retrieval using 0.01 mol/L sodium citrate containing 0.05% Tween. IHC was performed on paraffin-embedded mouse tissues using antibodies targeting FAP antibody (ABclonal, A23789), PD-L1 (absin, abs136046), Ki-67 (CST, 12202), CD4 (abcam, ab183685), CD8 (abcam, ab217344), GZB (abcam, ab4059), and Ly6G (CST, 87048S), in accordance with our previously established protocol utilizing the aforementioned monoclonal antibodies (mAb) (reference 40 in the manuscript). Immunofluorescence staining was performed employing antibodies against CD86 (to identify M1 macrophages, CST, 19589S) and CD206 (to identify M2 macrophages, R&D, AF2535). Each slide was then treated with two drops of DAPI (D1306; Thermo Fisher), washed in distilled water, and manually cover-slipped. The slides were then air-dried and mounted with Prolong Diamond Anti-fade mounting medium (P36965; Thermo Fisher). Pictures were taken with the Aperio Versa 8 tissue imaging system (3D HISTECH). Images was analysed using Indica Halo software. IHC staining was conducted. Immunofluorescent TUNEL analysis was performed using a commercially available kit (Beyotime, C1098). To evaluate the effect of treatment on normal organs, the main organs, including the heart, liver, lungs, kidneys, and spleen were collected for H&E staining.

*In vivo bioluminescence imaging*

For tumor implantation, the right rear flanks of C57BL/6 mice were injected with a 100 μL suspension. This suspension contained tumor cells (MC38, 1 × 10^6^ cells) mixed with fibroblasts (LUC-transfected NIH3T3-FAP cells, 2 × 10^6^ cells). On Day 12, *in vivo* bioluminescence imaging (BLI) was performed on mice anesthetized with Isoflurane, after administering D-Luciferin (150 mg/kg intraperitoneally, given 15–25 minutes prior to imaging), with an auto acquisition time at 37 °C using an IVIS Lumina III (PerkinElmer, USA).

*Single cell isolation from mice tumor tissue*

For the isolation of single cells from MC38/NIH3T3-FAP tumors, tissues were collected from mice in different treatment groups. Tumors were dissected and washed multiple times with pre-cold PBS to remove necrotic content. They were then minced into small pieces and incubated with 0.5 mg/mL digestive enzyme 1 (prepared by 10K Genomics) for 30 min at 37℃ in a water bath. After incubation, the mixture was centrifuged, and the supernatant was collected. The remaining pellet was subjected to a second round of digestion with 0.5 mg/ml digestive enzyme 1 for an additional 30 min. Following a total of 60 min of digestion, the supernatant and pellet were combined and filtered through a 70 μM cell strainer (BD Falcon, 352350). The mixture was then centrifuged at 500 g for 5 min. The resulting pellet was further digested with 1 mL of trypsin for 15 min. The supernatant was collected and passed through a 35 μM cell strainer (BD Falcon, 352235). After another centrifugation at 500 g for 5 min, the cell pellet was resuspended in PBS containing 0.01% BSA.

*Isolation of Human Peripheral Blood Single Nucleated Cells*

Approximately 5 mL of subject peripheral blood was collected using an EDTA blood collection tube. The collected blood was diluted with an equal volume of PBS and mixed gently. A 15 mL tube was filled with an equal volume of Ficoll (GE, 17144003), and the PBS-diluted blood was slowly added along the tube wall. After centrifugation at 700 g for 20 minutes, the white PBMC cell layer was transferred to another 15 mL tube. The solution volume was brought up to 10-15 mL with PBS, centrifuged at 500 g for 5 minutes, and the precipitate was resuspended in 1 mL of erythrocyte lysis buffer (BD, 555899) and incubated at room temperature for 5 minutes to remove the erythrocytes. The reaction was terminated by adding 10 mL of resuspension buffer (PBS+0.01% BSA), followed by centrifugation at 500 g for 5 minutes. The precipitate was then resuspended in the appropriate volume of resuspension buffer.

*Single Cell RNA-Seq*

PBMC suspensions were loaded onto a 10x Chromium Controller (10x Genomics) according to the manufacturer's instructions. Single-cell RNA-Seq libraries were prepared using the Chromium Single-Cell 3’-End V3.1 Kit (Cat. CG000204 Rev D). Briefly, single-cell suspensions were processed through a 10x Chromium Controller to form emulsions, and individual cells in each droplet were exposed to gel beads coupled with unique primers bearing 10X cell barcodes, unique molecular identifiers (UMIs), and poly(dT) sequences. Barcoded full-length cDNAs were generated using a reverse transcription reaction. Subsequently, the emulsion was broken, and the cDNAs were collected, amplified, and enriched using the reagents in the Chromium Single Cell 3' V3.1 Reagent Kit. Indexed sequencing libraries were constructed through the following steps: (1) fragmentation, end repair, and A-tailing; (2) size selection of cDNA fragments using SPRI beads; (3) ligation of sequencing adapters; (4) further enrichment of cDNA fragments with SPRI beads; and (5) indexing and final enrichment of libraries with SPRI beads. The amount of library to be sequenced was determined based on the number of cells and was sequenced on a NovaSeq 6000 (Illumina) using paired-end 150 bp reads.

*scRNA-seq Data Processing*

The scRNA-seq FASTQ files were processed using Cell Ranger software. hg38 (human) and mm10 (mouse) were used for genome alignment to obtain 10X expression matrix files for each sample. The 10X matrix file for each sample was further analyzed by the Seurat package (version 4.3.0) in R software (version 4.2.2). For each cell, four quality control (QC) measures were applied. Cells meeting any of the following criteria were excluded: (1) < 500 expressed genes, (2) percentage of mitochondrial genes > 10%, (3) total number of mRNA molecules < 1000 or > 20,000, (4) percentage of erythrocyte genes > 5%.

*Sample Integration, Dimensionality Reduction, and Clustering*

The gene expression matrix was normalized by the NormalizeData function using parameters scale.factor = 10000, normalization.method = "LogNormalize". The top 1000 highly variable genes were selected for principal component analysis (PCA) by the FindVariableFeatures function, and the top 20 principal components (PCs) were used in the FindNeighbors function. For cell clustering, we used the FindClusters function in Seurat, with the resolution parameter set to 0.5 for all cells (PBMC and mouse), while for subpopulations a resolution of 1 was used. RunUMAP was used to perform Uniform Manifold Approximation and Projection (UMAP) for 2D visualization, with the default parameters. With CellMarker (CellMarker (xbio.top)), we obtained typical cell markers for various cell lineages and used these markers to assign major cell lineages to each cell cluster.

*Differential Expression Analysis and Gene Set Variation Analysis (GSVA)*

Differential expression analysis was performed on cells from the seven-day post-treatment group and the response group using the FindAllMarkers function. To assign pathway activity estimates to each sample, we used gene set variation analysis as implemented in the GSVA R package (version 1.32.0). The GO gene sets for the immune-related pathways we studied were obtained from the Gene Set Enrichment Analysis (GSEA) website (https://www.gsea-msigdb.org/gsea/index.jsp). The R package heatmap was used to plot gene set scores between different subgroups.

*RNA Velocity Analysis*

First, the velocity software was used to generate a loom file from the sorted bam file and the genome annotation file "GRCmm10.gtf". After that, the loom files of each sample were merged into a single loom file. The R package sceasy was used to convert the cell Seurat files of each subpopulation into h5ad files, and we used the scanpy package of Python to generate the AnnData object, based on the scVelo algorithm, and the Python package scVelo for RNA velocity analysis to calculate the RNA velocity and plot the visualization.

*Monocle2*

We also used Monocle2 (version 2.14.0) to infer the cellular pedigree trajectory of neutrophils containing the top 1000 characterized genes with q-values < 0.001 calculated by the DifferentialGeneTest function. After downscaling and cell sorting, differentiation trajectories were inferred using Monocle's default parameters.

*Analysis of Cell-to-Cell Interactions*

We used CellChatDB.mouse in CellChat (version 1.6.1) to infer intercellular interactions between different cell types. The method infers the strength of potential interactions between two cell subpopulations based on gene expression levels, and we used the netVisual_bubble function to calculate receptor-ligand interactions.

*SCENIC Analysis*

Transcription factors in each tumor cell were analyzed using pySCENIC software with the raw count matrix as input. Co-expression networks were calculated by runGenie3, and regulators were identified by RcisTarget. AUCell scored each cell for regulator activity.

*TCR*

About 2 mg total RNA was isolated from each tumor sample utilizing the TRIzol reagent (Invitrogen) following the manufacturer's guidelines. Subsequently, an RNA sequencing library was meticulously prepared by employing the KC Stranded mRNA Library Prep Kit for Illumina (Wuhan Seqhealth Co., Ltd.), as per the kit instructions. The library was then sequenced using the advanced HiSeq X10 sequencer from Illumina. The low-quality reads were filtered out, and the remaining sequences were reserved for further analysis. IGBLAST software was used to find TCR V, D, and J genes in each read based on the TCR reference genome downloaded from the International Immunogenetics Information System (IMGT)/GeneDB database. Reads containing V, D, and J gene segments were extracted and further translated into CDR3 aa sequences. Jaccard index was calculated to assess the similarity of the TCR-β repertoire across different tumor tissues. This metric is calculated by dividing the number of overlapping clones by the total number of unique clones present in two samples, ranges from 0 to 1. Finally, the ggseqlogo 0.1 package was used to identify the motif of CDR3.

*Scintigraphy and SPECT/CT imaging*

The kinetics of ^177^Lu-LNC1004 were determined using planar whole-body scintigraphy in the anterior and posterior projections at 1-168 h after administration of the radiopharmaceutical using a double-head γ-camera (Symbia T16; Siemens, Erlangen, Germany) equipped with a low-energy high-resolution parallel-hole collimator and a 15% energy window set symmetrically over the 113 keV and 208 keV photopeaks. The table speed was 10 cm/min, and the matrix was 256 × 1024, producing 2.4 × 2.4 mm pixels. Furthermore, whole-body SPECT/CT was performed at 72 or 96 h after administration. CT was performed for attenuation correction without a contrast agent (tube voltage, 130 kVp; tube current-time product, 17 mAs; beam pitch, 1.5; section width, 5 mm), and SPECT scans were acquired at 128 angles over 360° and 25 s per stop. Images were iteratively reconstructed and corrected for attenuation and scatter (Flash 3D Siemens, 4 subsets and 8 iterations; Gaussian intersection smoothing filter; attenuation coefficient, 0.15/cm). The image matrix was 128 × 128, resulting in a cuboid voxel length of 4.8 mm.

*Clinical, radiologic, and laboratory follow-up*

Routine assessments, including complete blood counts, organ function tests, and tumor markers, were conducted biweekly. All patient records were meticulously reviewed for any adverse events (AEs), graded according to the CTCAE 5.0.


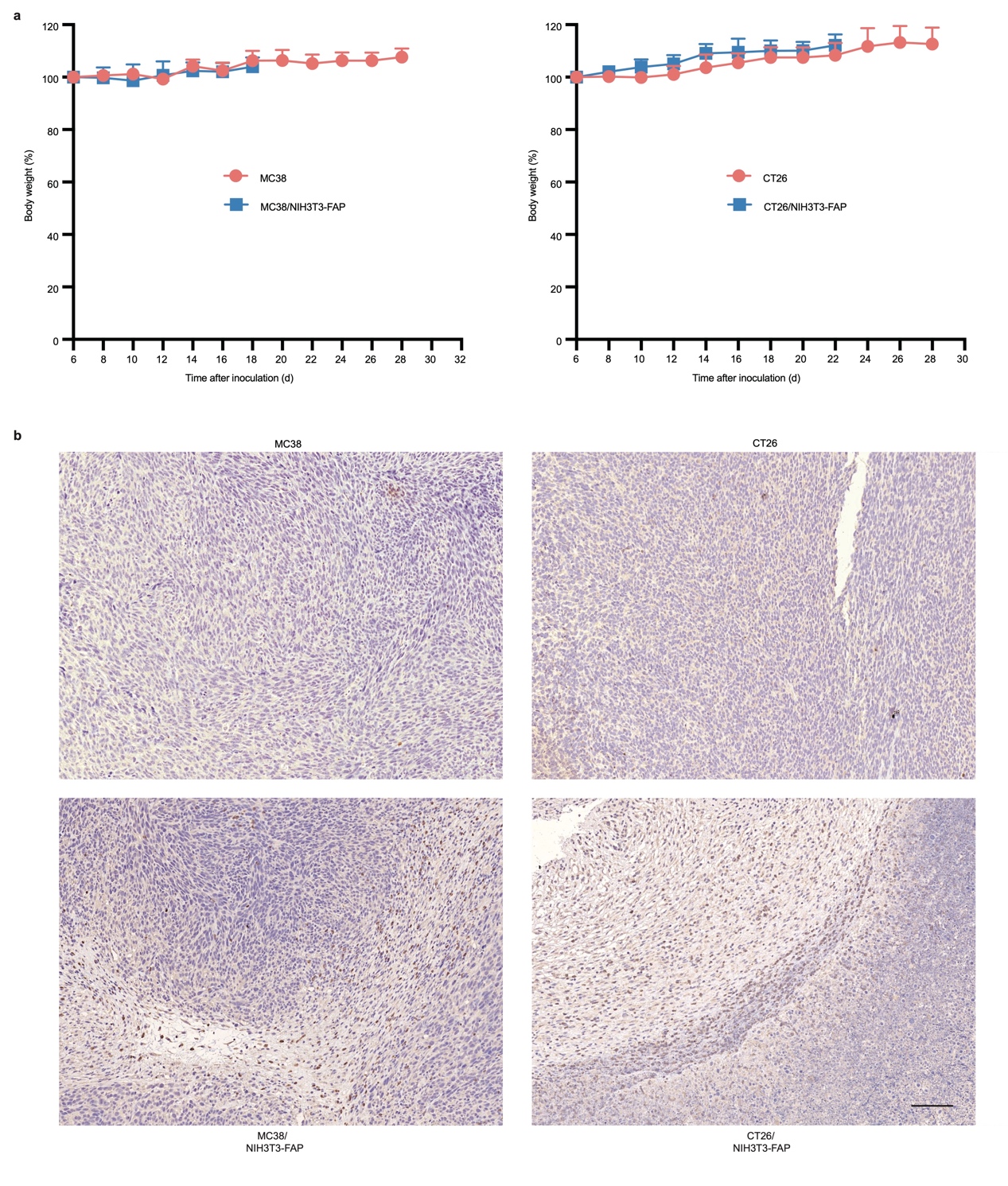


Figure S1. (a) Body weight change in MC38, MC38/NIH3T3-FAP, CT26, and CT26/NIH3T3-FAP. (b) Immunohistochemical staining of FAP in tumor tissues of MC38, MC38/NIH3T3-FAP, CT26, and CT26/NIH3T3-FAP tumors. Scale bar: 200 μm.


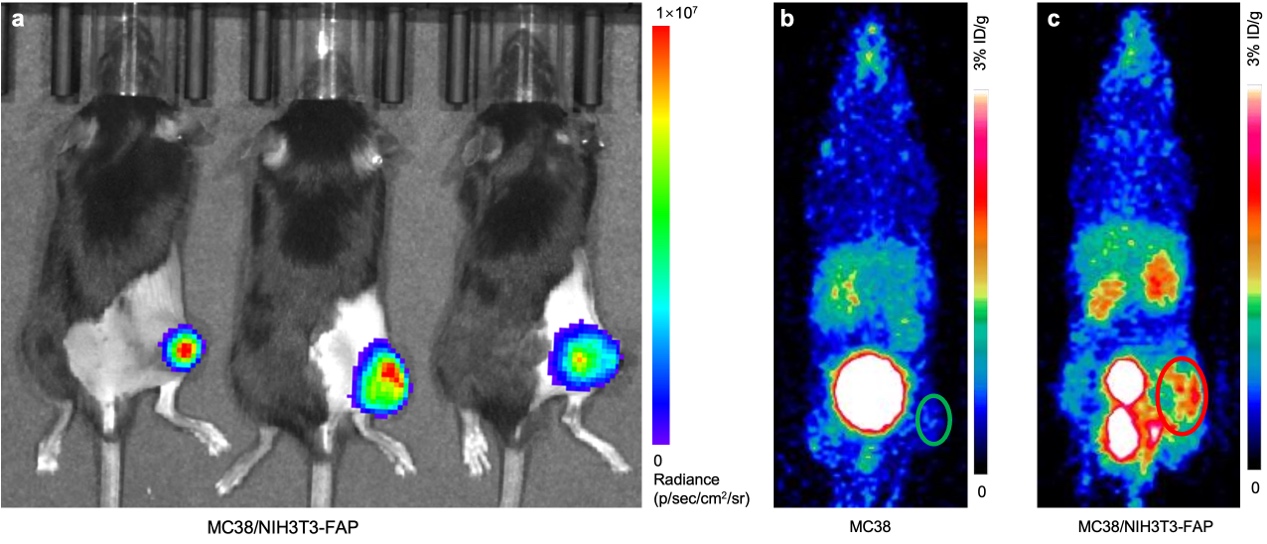


Figure S2. (a) Small animal fluorescence *in vivo* imaging was conducted on day 12 following the co-injection of LUC-transfected NIH3T3-FAP cells and MC38 cancer cells. Representative static PET images showing the distribution of ^68^Ga-FAPI-46 in mice bearing MC38 tumors (b) and MC38/NIH3T3-FAP tumors (c) were captured on the 12th day post cell injection. Areas encircled in the images denote tumor tissue.


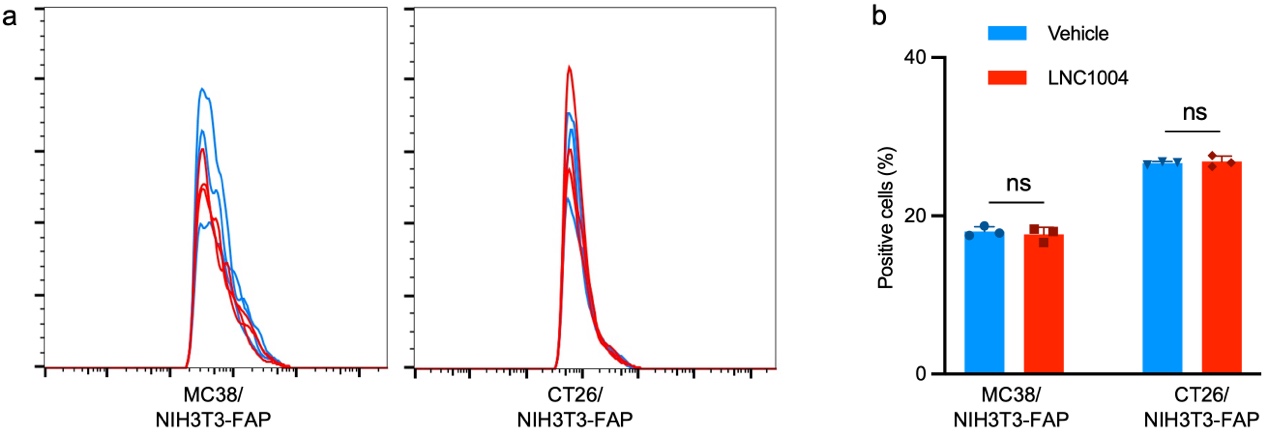
Figure S3. Representative histograms (a) and bar plot (b) derived from flow cytometry showing the regulation in PD-L1 expression after 24 h of stimulation with LNC1004 (n = 3/group).


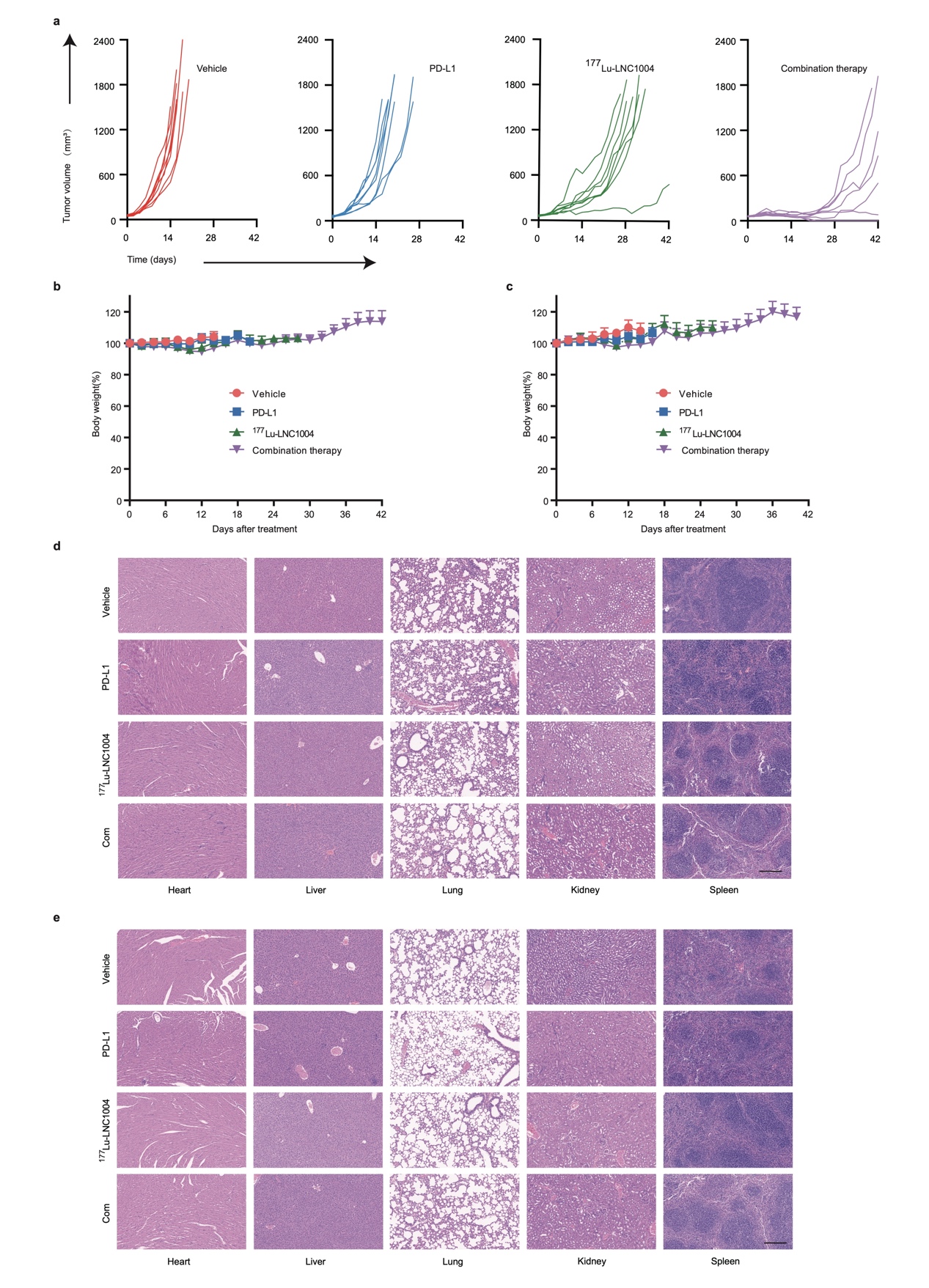


Figure S4. (a) Individual tumor growth trajectories are presented for CT26/NIH3T3-FAP tumor-bearing mice across various treatment groups. (b-c) Changes in body weight post-treatment are shown for both MC38/NIH3T3-FAP and CT26/NIH3T3-FAP tumor-bearing mice. (d-e) H&E staining of normal organs—including the heart, liver, lung, kidney, and spleen—is demonstrated on day 12 post-treatment for both MC38/NIH3T3-FAP (d) and CT26/NIH3T3-FAP (e) tumor-bearing mice. Scale bar: 200 μm.


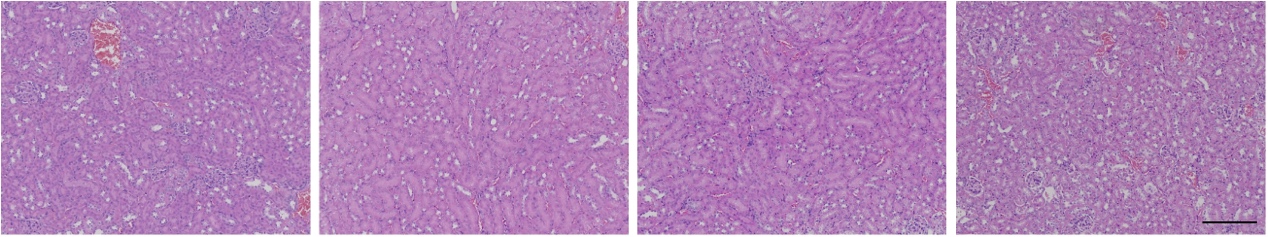


Figure S5. H&E staining of kidney is showed in MC38/NIH3T3-FAP tumor-bearing mice 8 months after the combination therapy with αPD-L1 and ^177^Lu-LNC1004 (n=4). Scale bar: 200 μm.


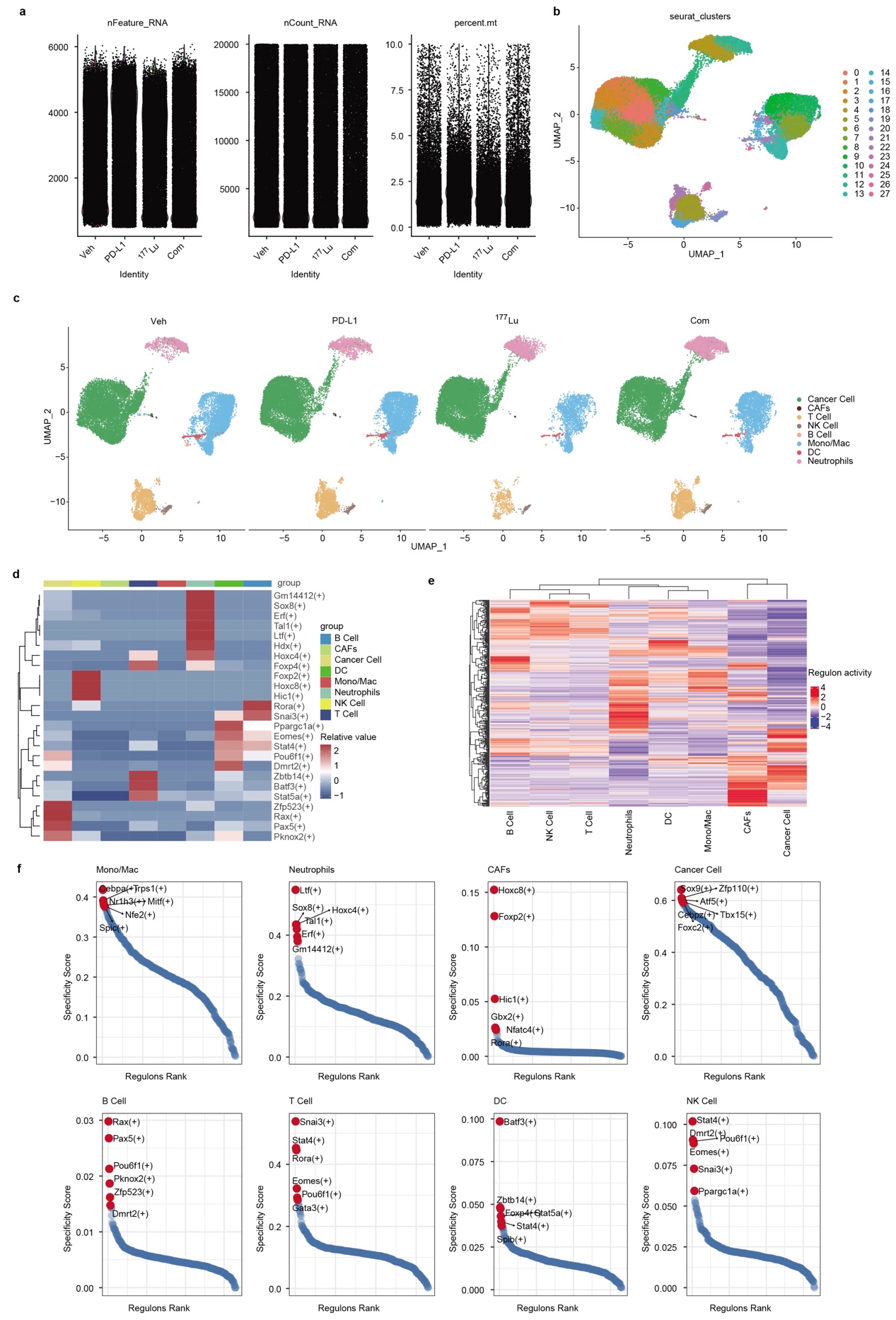


Figure S6. Sample quality control for single-cell sequencing, major lineage clustering, and transcription factor analysis. (a) QC results for single-cell sequencing samples. (b) UMAP plot of all cells categorized into 28 clusters. (c) UMAP plots of the cells from Veh (Vehicle), PD-L1 (PD-L1 treatment), ^177^Lu (^177^Lu-LNC1004 treatment) and Com (Combination therapy). (d) Heatmap shows differences in transcription factor AUC values for individual cells. (e) Heatmap shows differences in transcription factor expression values for individual cells. (f) Dot plots shows differences in transcription factor-specific scoring of individual cells.


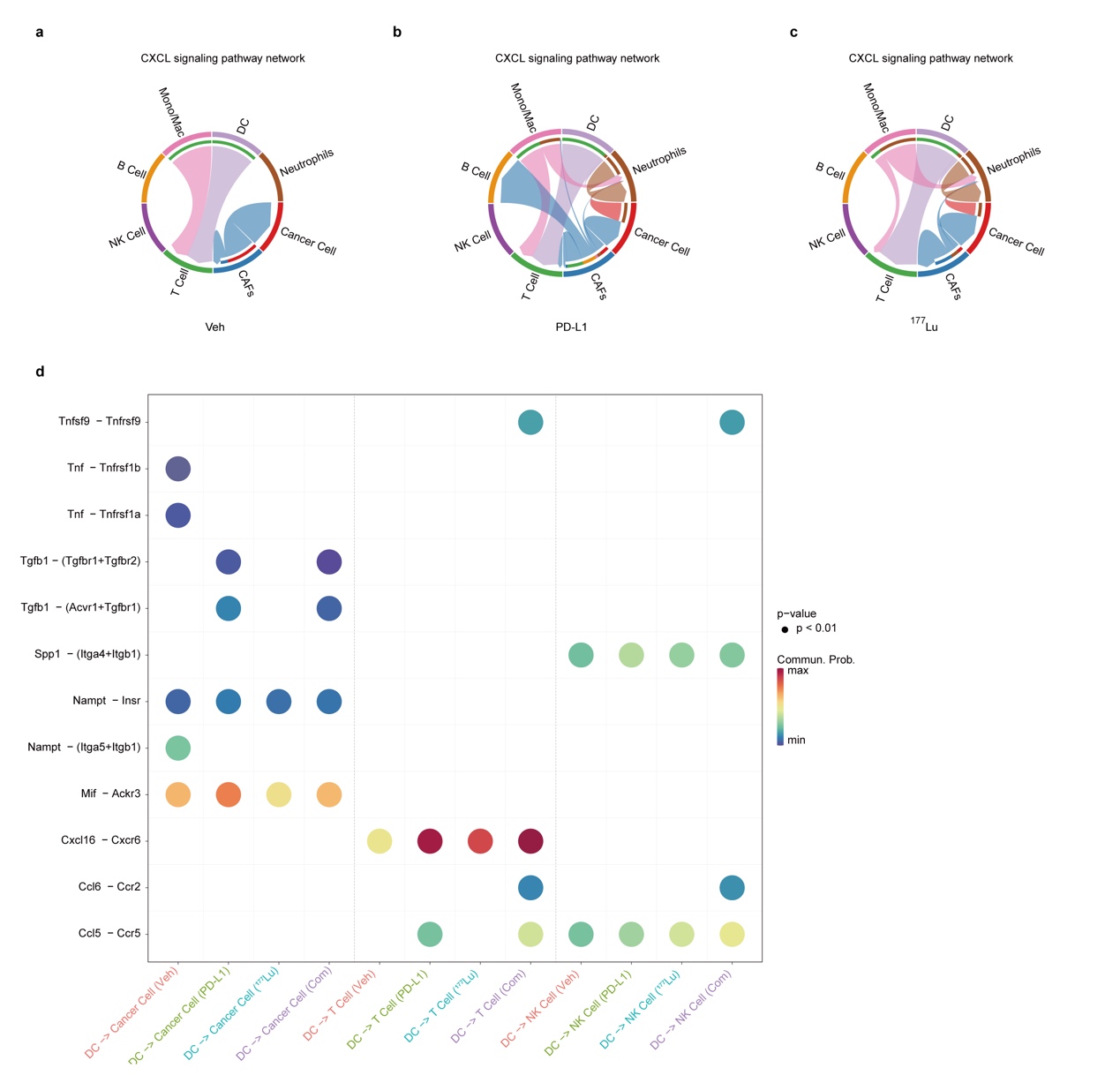


Figure S7. (a-c) Interaction networks emphasize specific cell-to-cell interactions in the CXCL pathways within the vehicle, PD-L1, and ^177^Lu-LNC1004 treatment groups. (d) Highlighted ligand-receptor interactions from DC to T cells, NK cells, and cancer cells, as informed by CellChat.


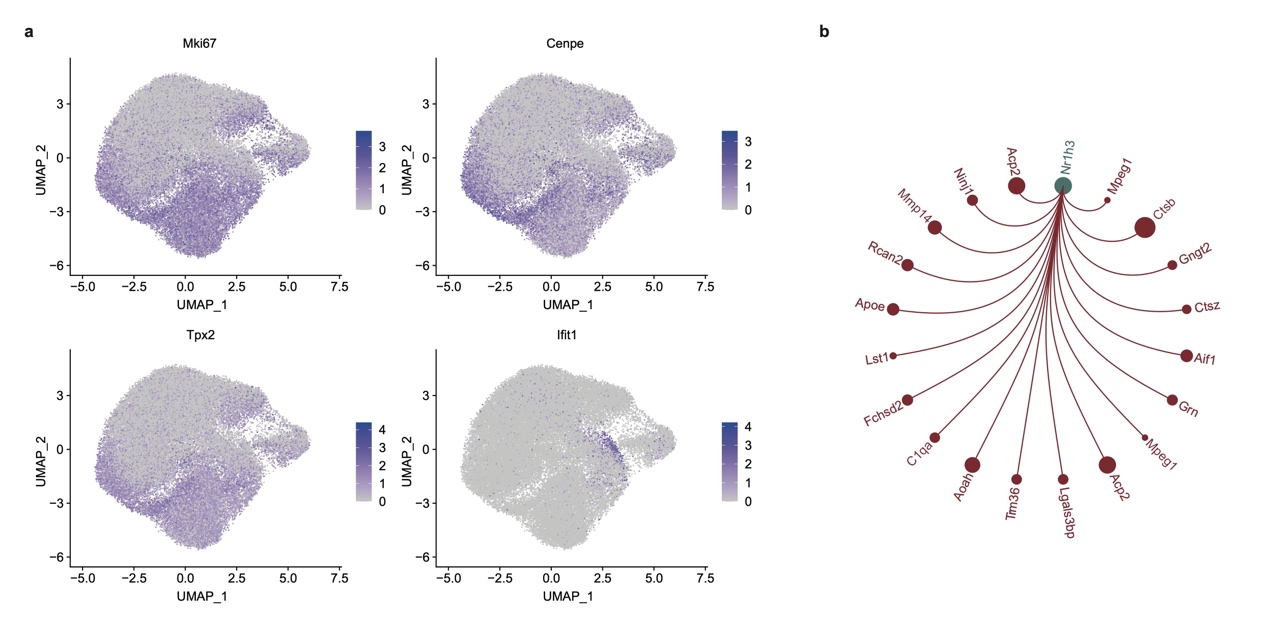


Figure S8. (a) UMAP plots showing the expression of selected marker genes for the defined cancer cell types. (b) Regulatory network diagram centered on transcription factors Nr1h3.


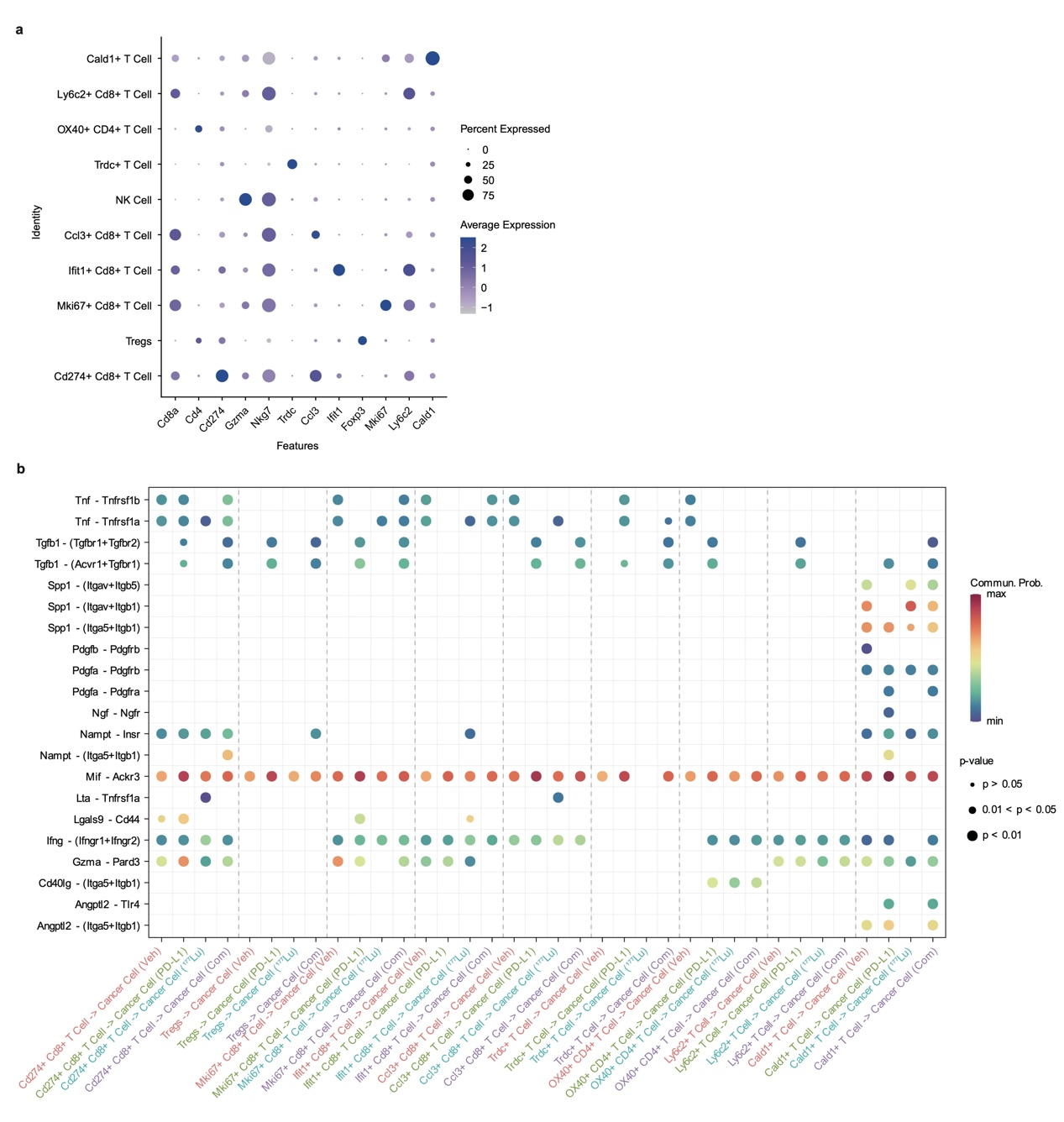


Figure S9. (a) Dot plots reveal characteristic markergene across different T cell fractions. (b) Highlighted ligand-receptor interactions from T cell subgroups to cancer cells, as informed by CellChat.


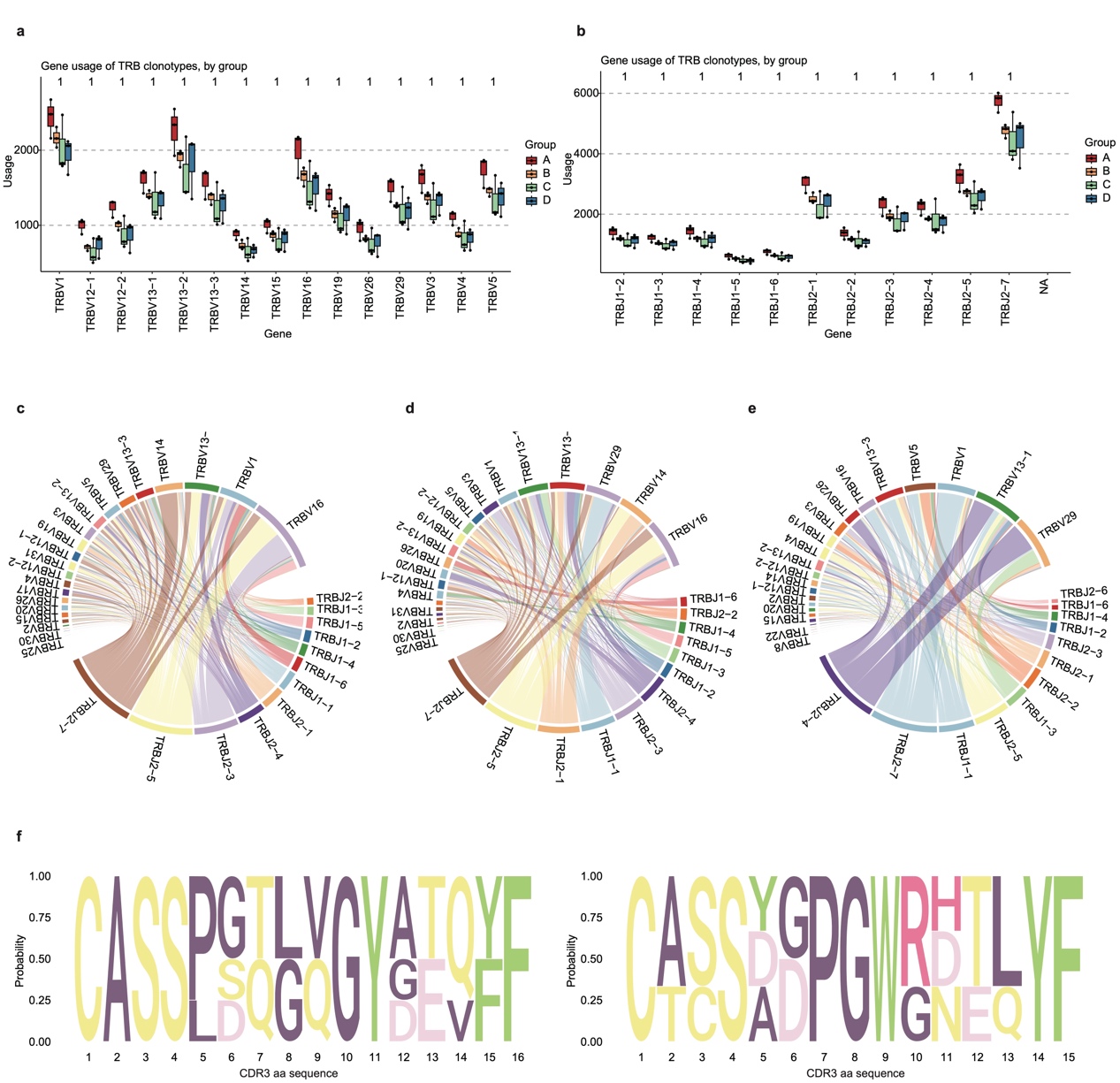


Figure S10. (a-b) Gene usage of TRB-V and TRB-J clonotypes, by group. The groups A-D correspond to the vehicle group, αPD-L1-treated group, ^177^Lu-LNC1004-treated group, and the combination therapy group with αPD-L1 and ^177^Lu-LNC1004, respectively. (c-e) Chord plots illustrate the frequency of distinct V-J gene pairings in the vehicle, PD-L1, and ^177^Lu-LNC1004 treatment groups. (f) Sequence logo diagrams present the CDR3 amino acid composition of the notably expanded TCR clonal family in the PD-L1 group (left) and in the ^177^Lu-LNC1004 treatment group (right).


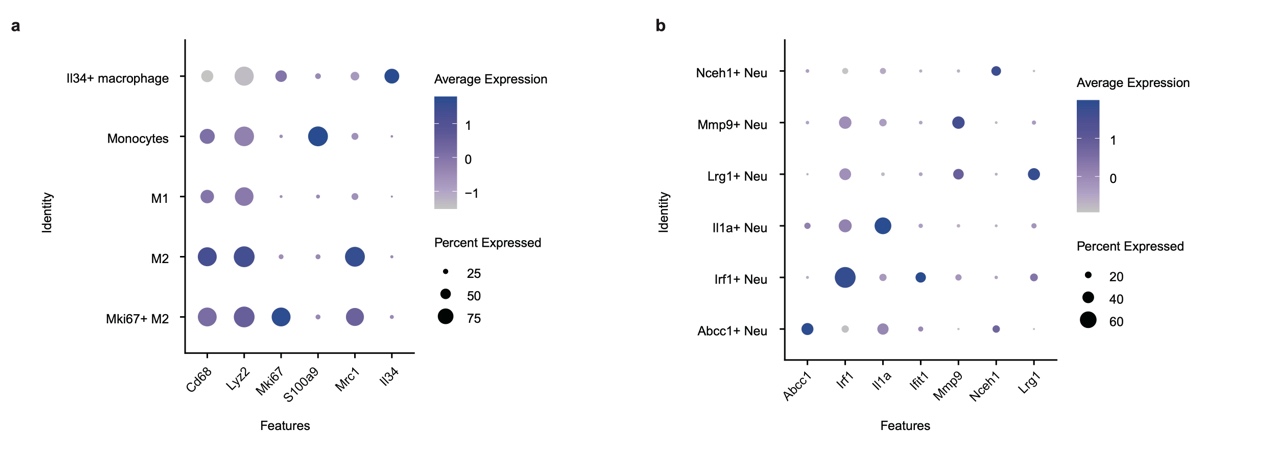


Figure S11. Dot plots reveal characteristic markergene across different Mono/Mac (a) and neutrophil cellular (b)fractions.


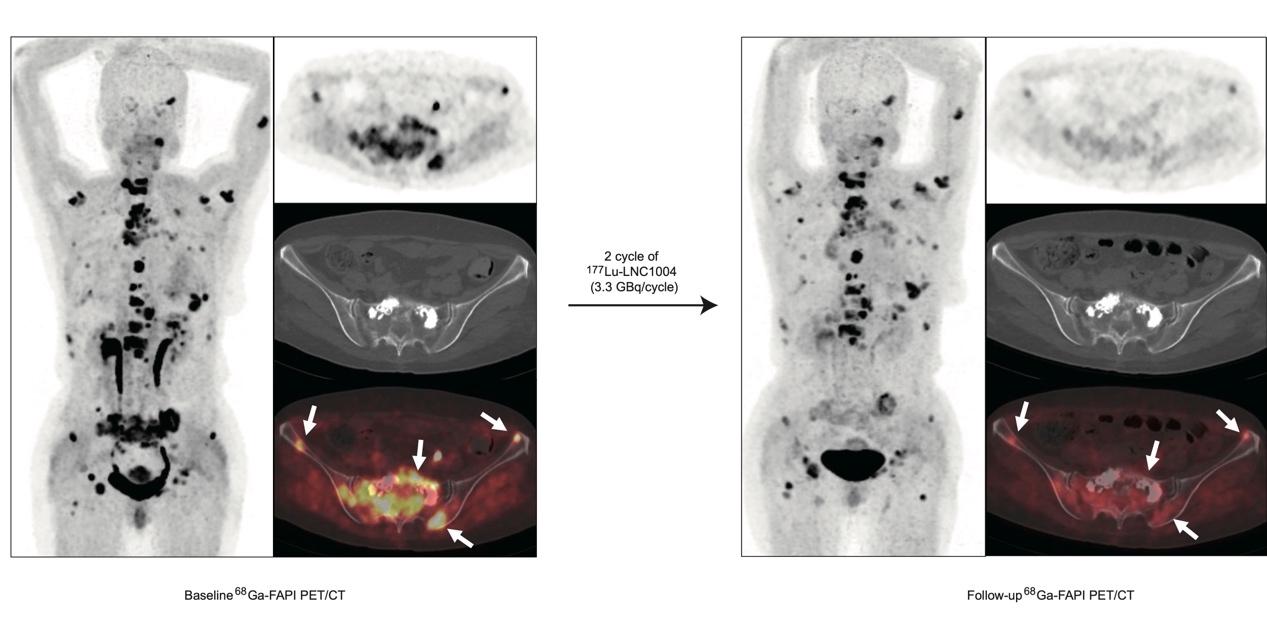


Figure S12. The representative case (Patient No.5) exhibited a favorable therapeutic response after 2 cycles of ^177^Lu-LNC1004. Baseline ^68^Ga-FAPI-46 PET/CT showed intense uptake in the widespread bone metastases (left). The follow-up ^68^Ga-FAPI-46 PET/CT revealed a significant reduction in both tumor size and radiotracer uptake in these metastatic lesions (right) after two cycles of ^177^Lu-LNC1004 therapy.


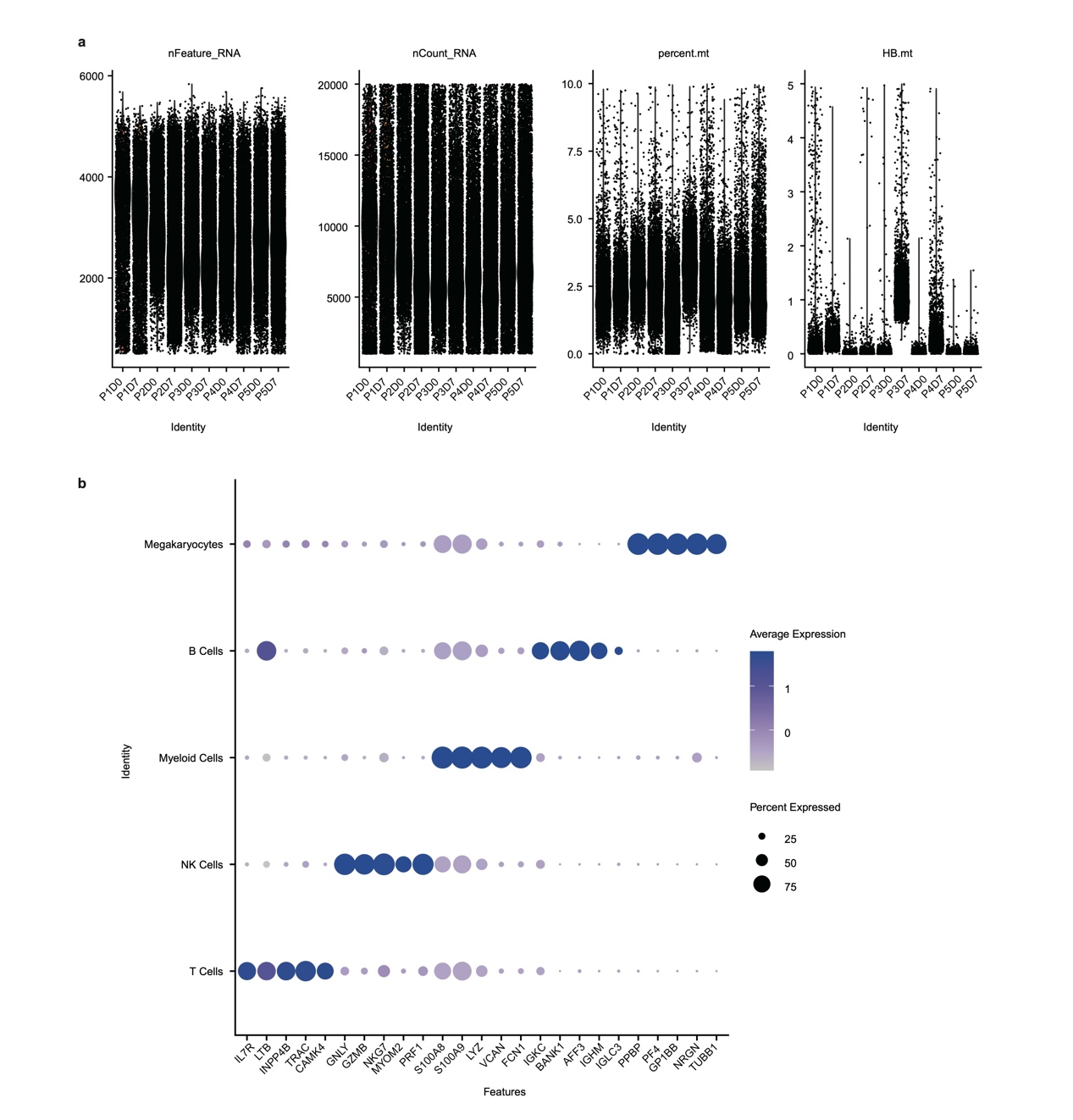
Figure S13. (a) Sample quality control for PBMC single-cell sequencing. (b) Dot plots reveal characteristic markergene across different cellular fractions.


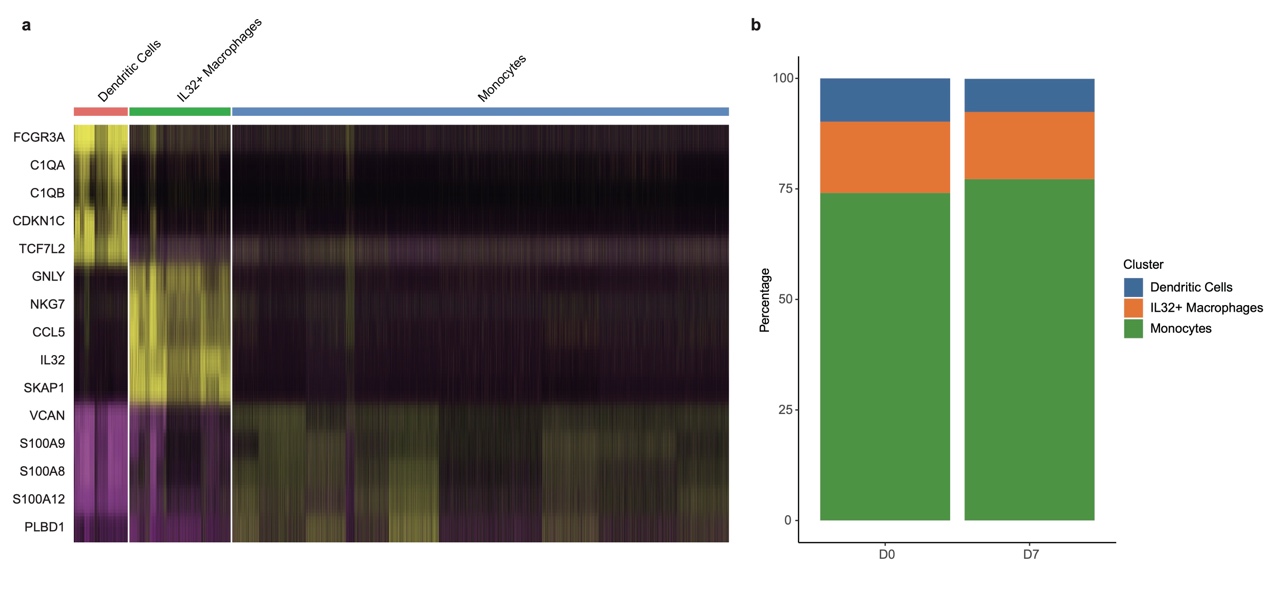


Figure S14. (a) Heatmap plots showing the expression of selected marker genes for the defined myeloid cell types. (b) Bar charts elucidate the variations in the proportions of myeloid cell subtypes across patients before and after therapeutic interventions.


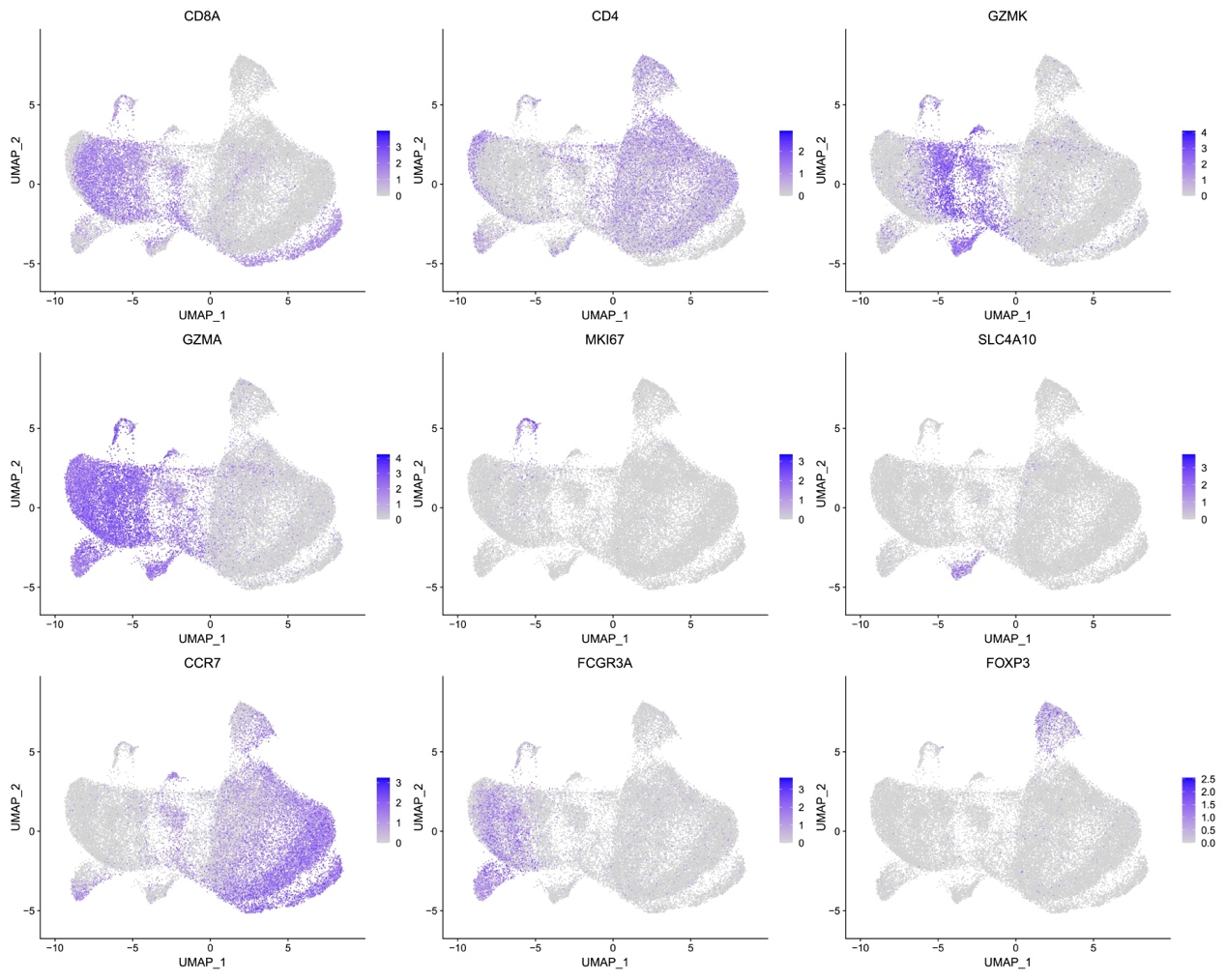


Figure S15. UMAP plots showing the expression of selected marker genes for the defined T cell types.

| **Table S1.** Adverse Events After Onset of Treatment, Related or Unrelated. | | | | | | | | | | | | | | | | | | | | | | | | | |
| --- | --- | --- | --- | --- | --- | --- | --- | --- | --- | --- | --- | --- | --- | --- | --- | --- | --- | --- | --- | --- | --- | --- | --- | --- | --- |
| Patient No. | General | Laboratory-based AEs | | | | | | | | | | | | | | | | | | | | | | |  |
|  |  | Hematology | | | | | | | | | | |  | Kidney | |  | Liver | | | | | | | |  |
|  |  | WBCs | |  | ANC | |  | Hb | |  | PLTs | |  | sCr | |  | T Bill | |  | AST | |  | ALT | | New G3/G4 AE (laboratory) |
|  |  | B | F |  | B | F |  | B | F |  | B | F |  | B | F |  | B | F |  | B | F |  | B | F |  |
| 1 | Tumor progression | **—** | **—** |  | **—** | **—** |  | G2 | G2 |  | **—** | **—** |  | **—** | **—** |  | **—** | **—** |  | **—** | **—** |  | **—** | **—** | No |
| 2 | Tumor progression | **—** | **—** |  | **—** | **—** |  | — | — |  | **—** | G3 |  | G1 | **—** |  | **—** | **—** |  | G1 | G1 |  | **—** | **—** | Yes |
| 3 | COVID-19 Pneumonia, fever | **—** | **—** |  | **—** | **—** |  | G1 | G1 |  | **—** | **—** |  | **—** | **—** |  | **—** | **—** |  | **—** | **—** |  | **—** | **—** | No |
| 4 | Tumor rupture, tumor progression | G1 | **—** |  | **—** | **—** |  | G1 | G1 |  | G1 | G1 |  | **—** | **—** |  | G3 | G3 |  | G2 | **—** |  | G1 | **—** | No |
| 5 | None | **—** | **—** |  | **—** | **—** |  | G2 | G2 |  | **—** | **—** |  | **—** | **—** |  | **—** | **—** |  | **—** | **—** |  | **—** | **—** | No |
|  | Any new AE (%) | **—** | |  | **—** | |  | **—** | |  | 1 (20%) | |  | **—** | |  | **—** | |  | **—** | |  | **—** | |  |
|  | Any new G3/G4 AE (%) | **—** | |  | **—** | |  | **—** | |  | 1 (20%) | |  | **—** | |  | **—** | |  | **—** | |  | **—** | |  |

AEs: adverse events, SD: stable disease, WBCs: white blood cells, ANC: absolute neutrophil count, Hb: hemoglobin, PLTs: platelets, sCr: serum creatinine, T bill: total bilirubin, AST: aspartate aminotransferase, ALT: aspartate aminotransferase, G1: grade 1, G2: grade 2, G3: grade 3, G4: grade 4, No.: number, B: baseline, F: follow-up.
